# Trends and outcomes of children, adolescents, and adults hospitalized with inherited metabolic disorders: A population‐based cohort study

**DOI:** 10.1002/jmd2.12320

**Published:** 2022-08-10

**Authors:** Stephanie Isabelle Hauser, Claudia Gregoriano, Henrik Koehler, Fahim Ebrahimi, Gabor Szinnai, Philipp Schuetz, Beat Mueller, Alexander Kutz

**Affiliations:** ^1^ Medical University Department of Medicine Kantonsspital Aarau Aarau Switzerland; ^2^ Department of Pediatrics Kantonsspital Aarau Aarau Switzerland; ^3^ Department of Clinical Research University Hospital Basel, University of Basel Basel Switzerland; ^4^ Division of Gastroenterology, University Center for Gastrointestinal and Liver Diseases St. Clara Hospital and University Hospital Basel Switzerland; ^5^ Department of Pediatric Endocrinology and Diabetology University Children's Hospital Basel Basel Switzerland

**Keywords:** amino acid disorders, carbohydrate disorders, clinical epidemiology, fatty acid disorders, inherited metabolic disorder, in‐hospital outcomes

## Abstract

Inherited metabolic disorders (IMDs) comprise a heterogeneous class of genetic disorders characterized by impaired biochemical functions in metabolism. However, incidences and outcomes of patients hospitalized with IMDs are largely unknown. We conducted a population‐based cohort study using nationwide in‐hospital claims data in Switzerland from 2012 to 2020. We assessed incidence rates of hospitalizations and hospital‐associated outcomes, stratified in five age groups (0–9, 10–19, 20–39, 40–59, and 60–90 years) and three types of IMDs (peptide, amine and amino acid metabolism disorders [AD], carbohydrate metabolism disorders [CD], fatty acid, and ketone body metabolism disorders [FD]). A total of 7293 hospitalizations with IMD were identified, of which 3638 had AD, 3153 CD, and 502 FD. Incidence rates for hospitalizations per 100 000 person‐years were highest under the age of 10 years across all types of IMDs (8.69 for AD, 5.73 for CD, 3.71 for FD) and decreased thereafter. In patients with AD and CD, hospitalization rates increased again in adults aged 60–90 years (7.28 for AD, 7.25 for CD), while they remained low in patients with FD (0.31). Compared to inpatients without IMD, adult IMD patients had a higher burden of hospital‐associated adverse outcomes including an increased risk of in‐hospital mortality, intensive care unit admission, mechanical ventilation, and longer length of hospital or intensive care unit stay. Incremental risk of 30‐day, 1‐year, and 2‐year hospital readmission was highest among children and adolescents with IMD.

**SYNOPSIS**

While highest incidence rates of hospitalizations with inherited metabolic disorders were found among young children and adults over 60 years, the incremental risk of hospital‐associated adverse outcomes was highest among adults.

## 1 INTRODUCTION

Inherited metabolic disorders (IMDs), also known as “Inborn Errors of Metabolism,” are a group of disorders characterized by impaired biochemical functions in metabolism, causing a deficient end product or an accumulation of an intermediate substrate. This results in a wide variety of clinical phenotypes.^1^ Typically, neonates present healthy at birth, with signs of IMD developing hours to days after birth.^2^ However, there has been an increasing awareness of late‐onset IMDs in adolescents and adults.^3^

Although individual IMDs are rare, collectively, they are marginally common with a reported global birth prevalence of approximately 50.9 in 100 000 births.^4^ The first IMD was described by Sir Archibald Garrod in 1902^5^ and in the 120 years since then, the number of unique IMDs has been expanding rapidly with most recent classifications describing 1450 individual disorders.^6^ Additionally, the number of persons diagnosed with an IMD has been increasing throughout the years, presumably due to the widespread implementation of newborn screening programs.^7^ Thus, the number of patients living with IMDs is expanding, requiring an increasing need for general and specialty services for these patients throughout all age groups.

IMD hospitalizations typically require urgent care and an interdisciplinary comprehensive in‐hospital work‐up to avoid major complications, such as developmental delays, acute or chronic encephalopathy, and liver failure.^1^ While important progress regarding identification and management of IMDs has been made, knowledge of epidemiological trends and in‐hospital adverse outcomes of hospitalizations with IMD across the whole age spectrum remains poorly explored.

Hence, in this nationwide cohort study, we first aimed to investigate incidence rates of hospitalizations with IMD across the lifespan, stratified by main groups of IMDs, and second, to assess clinical outcomes of patients with IMD compared to those without IMD. This will allow an improved understanding of the burden of disease among hospitalized patients with IMDs.

## 2 METHODS

### 2.1 Study design

This analysis was conducted using a nationwide cohort of hospitalizations with IMD in Switzerland between 2012 and 2020. Hospitalization data were provided by the Swiss Federal Statistical Office (Neuchâtel, Switzerland), based on nationwide compulsory full census of Swiss hospitals. The dataset includes all Swiss inpatient discharge records from acute care‐, general‐, and specialty hospitals for both pediatric and adult patients. Individual‐level data on patient demographics, healthcare utilization, hospital typology, medical diagnoses, diagnostic tests, clinical procedures, and in‐hospital patient outcomes were provided. Multistep anonymization procedure ensured patient confidentiality and a unique patient identifier was used to ascertain rehospitalizations. Medical diagnoses were coded using the International Classification of Disease version 10, German Modification (ICD‐10‐GM) codes. Open‐source census data from the Swiss Federal Statistical Office on the Swiss population size, stratified by age and year, were obtained to calculate population‐based incidence rates of hospitalization with IMD. The institutional review board of Northwestern and Central Switzerland (EKNZ) waived the need for an ethical authorization due to the use of exclusively anonymized data (EKNZ Project‐ID: Req‐2021‐01397). This study adheres to the “Strengthening The Reporting of OBservational Studies in Epidemiology (STROBE)” statement.^8^

### 2.2 Case ascertainment and study variables

For this analysis, we included all hospitalizations of pediatric, adolescent, and adult persons in acute inpatient care, up to an age of 90 years, with identifiable IMD. Birth‐related hospitalizations of newborns were excluded. Hospitalizations with diagnosis codes for multiple groups of IMDs, where distinct classification was not possible, were also excluded. To identify IMDs, we used the International Classification of Inherited Metabolic Disorders online tool (ICIMD),^6^ specifically the group of “Intermediary Metabolism: Nutrients.” Each genetic disorder was matched with the corresponding ICD‐10‐GM code through the linked Orpha.net website^9^ wherever such information was available. The ICIMD subgroups “Peptide and Amine Metabolism Disorders” and “Amino Acid Metabolism Disorders” were combined to the group of “Peptide, Amine and Amino Acid Metabolism Disorders” (AD) and identified using ICD‐10‐GM codes E70.‐, E71.‐, and E72.‐, excluding E71.3. “Fatty Acid and Ketone Body Metabolism Disorders” (FD) were identified using E71.3. “Carbohydrate Metabolism Disorders” (CD) were identified using E74.‐ and E73.0. Comorbidities and cause of hospitalization were similarly identified using ICD‐10‐GM codes. The details on all ICD‐10‐GM codes used for the analysis are summarized in Table S1.

### 2.3 Outcomes

The primary outcome was the incidence rate of hospitalization with IMD per 100 000 person‐years and accompanying 95% confidence intervals (CI). Secondary outcomes comprised the occurrence of the following clinical endpoints: all‐cause in‐hospital mortality, total length of hospital stay (LOS, defined as days spent in the hospital during the hospitalization), intensive care unit (ICU) admission rate, length of ICU stay in days, intubation rate, and 30‐day, 1‐year, and 2‐year all‐cause hospital readmission rates. Lastly, cause of hospitalization was presented by proportions using given ICD‐10‐GM chapters. Cause of hospitalization was defined as the primary discharge diagnosis or secondary discharge diagnosis in case of IMD as primary discharge diagnosis. Analyses were stratified by age categories and group of IMDs.

### 2.4 Statistical analysis

Descriptive statistics were calculated for patient demographic information, including age, gender, and nationality. All baseline data are expressed as mean (standard deviation [SD]), median (interquartile range [IQR]), or number (%). Graphical depiction of hospitalization incidence rate per age was performed using locally estimated scatterplot smoothing (LOESS). Incidence rates (IR), incidence rate differences (IRD), and incidence rate ratios (IRR) per 100 000 person‐years with accompanying 95% CI were calculated for each age group, with FD as a reference. We used the Wald test for homogeneity to identify potential trend heterogeneity across IMDs groups.

To assess differences in hospital‐associated outcomes between patients with and without IMD, we first performed multivariable linear or logistic regressions as appropriate. The control group comprised hospitalized patients for any cause of acute care hospitalization without IMD. The models were adjusted for gender, age, year of admission, and main comorbidities as shown in the baseline characteristics table. To further increase the interval validity of the results, we performed a propensity‐score matched model. Eligible hospitalizations with IMD were 1:1 propensity‐score matched to a general medical inpatient cohort (matched controls) stratified by age category. The probability of having an IMD versus not having a coded IMD was calculated through a multivariable logistic regression model that contained all baseline covariates. The estimated propensity‐score was used to match IMD hospitalizations with nearest neighbor non‐IMD hospitalization with a caliper size of 0.01 on the propensity scale. Covariate balance before and after propensity‐score matching was assessed using standard differences. A standardized difference of less than 10% was considered as an adequate balance between groups.^10^ After propensity‐score matching, estimates of the effect sizes and corresponding 95% CI were determined using univariable linear or logistic regressions as appropriate. For subgroup analyses, we performed separate propensity‐score matchings per age group within each group of IMDs.

All *p*‐values are two‐sided and have not been adjusted for multiple testing. Results were considered statistically significant at *p* < .05. Statistical analyses were performed with STATA 17.0 (STATA Corp., College Station, TX).

## 3 RESULTS

### 3.1 Patient characteristics

**FIGURE 1:**
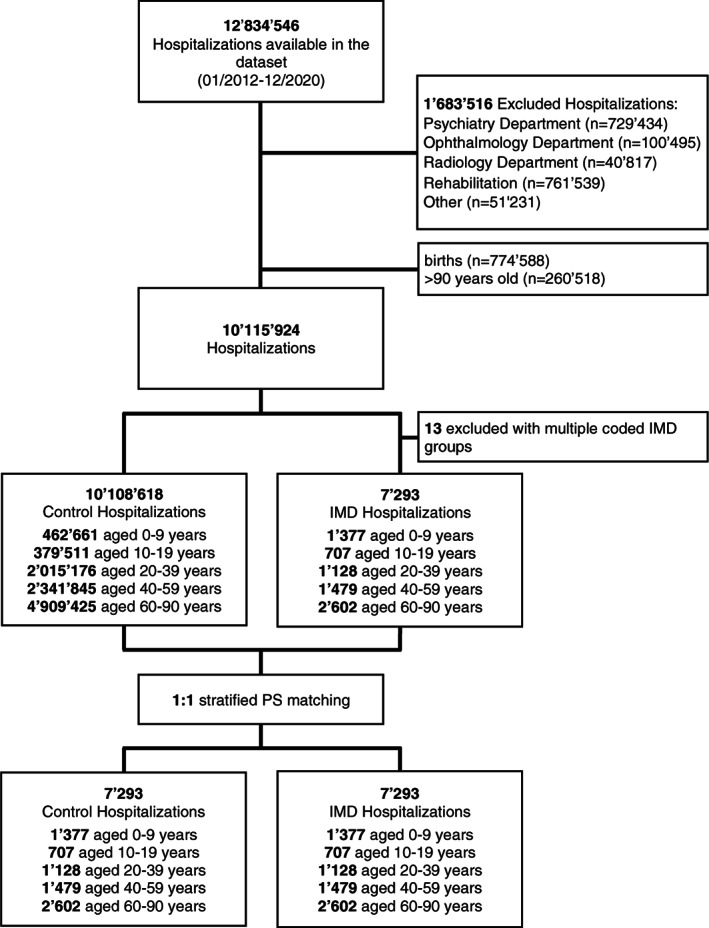
Study flowchart. IMD, inherited metabolic disorder; PS, propensity score

The full dataset included 12 834 546 hospitalizations between January 2012 and December 2020 (Figure 1). Of those, 7293 hospitalizations were identified with an IMD diagnosis, of which 3638 (49.9%) had a peptide, amine or amino acid metabolism disorder (AD), 502 (6.9%) a fatty acid and ketone body metabolism disorder (FD), and 3153 (43.2%) a carbohydrate metabolism disorder (CD). Disorders of urea cycle metabolism contributed most with 19.9% of hospitalizations (Table S1A). Overall, mean age was 42.5 years and 50.5% were female. In patients aged 0–9 years, main comorbidities were infectious and parasitic diseases, whereas the oldest age group showed an overall high burden of comorbidities. The characteristics of the study population stratified by age group are shown in Table 1. Patient characteristics after propensity score matching were well balanced and shown in Table S2.

**TABLE 1:** Baseline characteristics

	Overall	Age 0–9 years	Age 10–19 years	Age 20–39 years	Age 40–59 years	Age 60–90 years
Number of hospitalizations, *n*	7293	1377	707	1128	1479	2602
Number of individual patients, *n*	4910	587	345	732	1183	2152
*Socio‐demographics*						
Age, mean (SD)	42.5 (27.7)	2.5 (2.7)	14.6 (2.8)	29.6 (5.7)	50.4 (5.7)	72.4 (7.7)
Female gender, *n* (%)	3684 (50.5)	651 (47.3)	397 (56.2)	720 (63.8)	661 (44.7)	1255 (48.2)
Swiss nationality, *n* (%)	5345 (73.3)	834 (60.6)	463 (65.5)	736 (65.2)	1096 (74.1)	2216 (85.2)
*Type of inherited metabolic disorder, n (%)*						
Disorders of peptide, amine and amino acid metabolism	3638 (49.9)	660 (47.9)	343 (48.5)	574 (50.9)	785 (53.1)	1276 (49.0)
Disorders of fatty acid and ketone body metabolism	502 (6.9)	282 (20.5)	79 (11.2)	47 (4.2)	39 (2.6)	55 (2.1)
Disorders of carbohydrate metabolism	3153 (43.2)	435 (31.6)	285 (40.3)	507 (44.9)	655 (44.3)	1271 (48.8)
*Comorbidities, n (%)*						
Infectious and parasitic	2144 (29.4)	503 (36.5)	196 (27.7)	267 (23.7)	397 (26.8)	781 (30.0)
Neoplasms	841 (11.5)	33 (2.4)	38 (5.4)	51 (4.5)	199 (13.5)	520 (20.0)
Endocrine and metabolic	3833 (52.6)	353 (25.6)	277 (39.2)	440 (39.0)	872 (59.0)	1891 (72.7)
Mental and behavioral	1785 (24.5)	80 (5.8)	140 (19.8)	205 (18.2)	536 (36.2)	824 (31.7)
Nervous system	1817 (24.9)	211 (15.3)	148 (20.9)	173 (15.3)	431 (29.1)	854 (32.8)
Circulatory system	3149 (43.2)	182 (13.2)	88 (12.4)	184 (16.3)	722 (48.8)	1973 (75.8)
Respiratory system	1854 (25.4)	423 (30.7)	159 (22.5)	163 (14.5)	361 (24.4)	748 (28.7)
Digestive system	2588 (35.5)	187 (13.6)	179 (25.3)	289 (25.6)	686 (46.4)	1247 (47.9)
Musculoskeletal system	1273 (17.5)	43 (3.1)	72 (10.2)	151 (13.4)	257 (17.4)	750 (28.8)
Genitourinary system	2508 (34.4)	191 (13.9)	110 (15.6)	340 (30.1)	591 (40.0)	1276 (49.0)
Injuries	1416 (19.4)	135 (9.8)	83 (11.7)	196 (17.4)	357 (24.1)	645 (24.8)
Elixhauser comorbidities index, median (IQR)	2 (0, 3)	0 (0, 1)	1 (0, 1)	1 (0, 2)	2 (1, 4)	3 (2, 5)
Hospital frailty score						
<5 points	5305 (72.7)	1215 (88.2)	582 (82.3)	959 (85.0)	1067 (72.1)	1482 (57.0)
5–15 points	1717 (23.5)	157 (11.4)	123 (17.4)	156 (13.8)	347 (23.5)	934 (35.9)
>15 points	271 (3.7)	5 (0.4)	2 (0.3)	13 (1.2)	65 (4.4)	186 (7.1)

Abbreviation: IQR, interquartile range.

### 3.2 Hospitalization rates of patients with IMD

**FIGURE 2:**
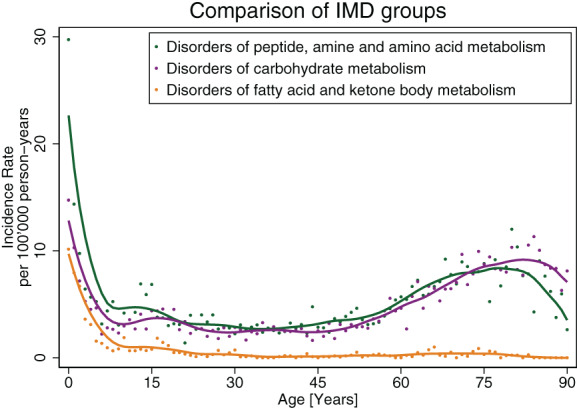
Trends of hospitalization of patients with IMD. Graphical depiction of incidence rates per 100 000 person‐years for hospitalizations of patients with peptide, amine and amino acid metabolism disorders (green), fatty acid and ketone body metabolism disorders (orange), and carbohydrate metabolism disorders (purple), shown over patient age using locally estimated scatterplot smoothing (LOESS). IMD, inherited metabolic disorder

**FIGURE 3:**
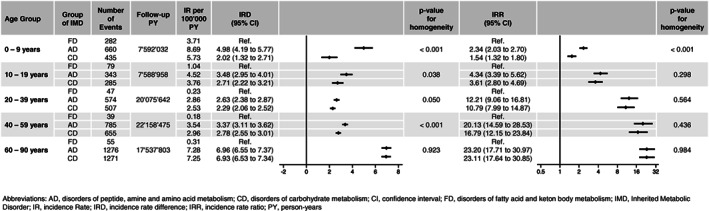
Difference in absolute and relative risk between IMD groups per age group. Graphical depiction of incidence rate differences (IRD) and incidence rate ratios (IRR) as forest plots, the latter on a natural logarithmic scale. IRD and IRR of AD and CD per age group, with FD as reference, with corresponding 95% CI and *p*‐values for homogeneity, were calculated. AD, disorders of peptide, amine and amino acid metabolism; CD, disorders of carbohydrate metabolism; CI, confidence interval; FD, disorders of fatty acid and ketone body metabolism; IMD, inherited metabolic disorder; IR, incidence rate; IRD, incidence rate difference; IRR, incidence rate ratio; PY, person‐years

In all three groups of IMDs, rates of hospitalization were highest among young children aged 10 years and below, showing incidence rates of 8.69, 3.71, and 5.73 per 100 000 person‐years for AD, FD, and CD, respectively. There was a steep decline of rates throughout childhood in all groups, with rates between 2.5 and 4.5 for AD and CD, and between 0.18 and 1.04 for FD, up to the age of 60 (Figures 2 and 3). While in the oldest age group hospitalization rates for FD remained low (0.31 per 100 000 person‐years), there was an increase to 7.28 and 7.25 cases per 100 000 person‐years in AD and CD, respectively. Throughout all age groups, IRD and IRR between AD or CD and FD were different, with highest rate differences among patients aged 60–90 years (AD vs. FD: 6.96 [95% CI, 6.55–7.37]; CD vs. FD: 6.93 [95% CI, 6.53–7.34]). Similar, incidence rate ratios were highest among the oldest population using FD as a comparator (AD vs. FD: 23.20 [95% CI, 17.71–30.97]; CD vs. FD: 23.11 [95% CI, 17.64–30.85]). Except for the youngest patients, when compared with FD on a relative risk scale, trends for hospitalization with AD and CD were similar across all age groups with no evidence for effect modification (*p* > 0.05) (Figure 3).

### 3.3 Hospital associated adverse outcomes

After the age of 19 years, when comparing with the overall non‐IMD inpatient population, most of the analyzed in‐hospital outcomes (in‐hospital mortality, longer length of hospital stay, ICU admission, longer length of ICU stay, mechanical ventilation) were more common among hospitalizations with IMD. In detail, for in‐hospital mortality, risk ratios for IMD hospitalizations ranged from 1.51 to 1.92, for ICU admission from 1.50 to 1.64, and for mechanical ventilation from 1.83 to 2.05. Length of hospital stay was prolonged by 2.73–3.31 days and length of ICU stay by 2.45–4.72 days. Results remained robust among propensity score matched cohorts (Table 2). Subgroup analyses exploring differences among specific groups of IMDs revealed highest risk of in‐hospital mortality in patients with AD across all age‐groups. Risk distribution for the remaining in‐hospital outcomes was similar to the overall analysis, with, in general, higher risks in the older population (Table S3A–E).

**TABLE 2:** Hospital associated adverse outcomes in patients with IMD by age group

		Age 0–9 years			Age 10–19 years			Age 20–39 years			Age 40–59 years			Age 60–90 years		
		Overall controls (*n* = 462 661)	1:1 PS matched controls (*n* = 1377)	IMD (*n* = 1377)	Overall controls (*n* = 379 511)	1:1 PS matched controls (*n* = 707)	IMD (*n* = 707)	Overall controls (*n* = 2 015 176)	1:1 PS matched controls (*n* = 1128)	IMD (*n* = 1128)	Overall controls (*n* = 2 341 845)	1:1 PS matched controls (*n* = 1479)	IMD (*n* = 1479)	Overall controls (*n* = 4 909 425)	1:1 PS matched controls (*n* = 2602)	IMD (*n* = 2602)
In‐hospital mortality	Events, *n* (%)	1225 (0.3)	13 (0.9)	23 (1.7)	469 (0.1)	4 (0.6)	11 (1.6)	2649 (0.1)	9 (0.8)	12 (1.1)	20 884 (0.9)	43 (2.9)	86 (5.8)	168 793 (3.4)	146 (5.6)	197 (7.6)
	Adjusted RR (95% CI)	Ref.		1.82 (1.20 to 2.76)	Ref.		2.05 (1.06 to 3.97)	Ref.		1.51 (0.86 to 2.67)	Ref.		1.92 (1.54 to 2.39)	Ref.		1.56 (1.37 to 1.79)
	RR (95% CI) after PSM		Ref.	1.77 (0.90 to 3.48)		Ref.	2.75 (0.88 to 8.60)		Ref.	1.33 (0.56 to 3.15)		Ref.	2.00 (1.40 to 2.86)		Ref.	1.35 (1.10 to 1.66)
Length of hospital stay (d)	Mean (SD)	4.0 (10.1)	7.7 (19.1)	7.6 (14.9)	3.6 (16.9)	6.2 (12.2)	7.8 (14.6)	3.8 (12.2)	6.5 (12.5)	8.6 (19.8)	4.7 (8.8)	9.3 (18.7)	11.4 (18.0)	7.5 (10.5)	10.5 (12.1)	13.7 (20.0)
	Adjusted Cf. (95% CI)	Ref.		1.05 (0.54 to 1.55)	Ref.		1.28 (0.05 to 2.52)	Ref.		3.06 (2.35 to 3.76)	Ref.		2.73 (2.30 to 3.15)	Ref.		3.31 (2.93 to 3.68)
	Cf. (95% CI) after PSM		Ref.	−0.13 (−1.41 to 1.16)		Ref.	1.53 (0.12 to 2.94)		Ref.	2.09 (0.72 to 3.46)		Ref.	2.10 (0.77 to 3.42)		Ref.	3.19 (2.29 to 4.09)
ICU admission	Events, *n* (%)	70 963 (15.3)	347 (25.2)	324 (23.5)	34 905 (9.2)	137 (19.4)	162 (22.9)	138 270 (6.9)	201 (17.8)	269 (23.9)	259 092 (11.1)	349 (23.6)	470 (31.8)	673 744 (13.7)	575 (22.1)	785 (30.2)
	Adjusted RR (95% CI)	Ref.		1.06 (0.96 to 1.19)	Ref.		1.35 (1.15 to 1.59)	Ref.		1.64 (1.44 to 1.87)	Ref.		1.51 (1.38 to 1.65)	Ref.		1.50 (1.40 to 1.60)
	RR (95% CI) after PSM		Ref.	0.93 (0.82 to 1.07)		Ref.	1.18 (0.97 to 1.45)		Ref.	1.34 (1.14 to 1.58)		Ref.	1.35 (1.20 to 1.52)		Ref.	1.37 (1.24 to 1.50)
Length of ICU stay (d)	Mean (SD)	3.2 (11.7)	7.5 (26.4)	6.0 (16.0)	1.2 (4.5)	3.1 (9.3)	4.6 (12.9)	1.0 (4.3)	3.7 (14.4)	5.8 (21.4)	1.7 (4.8)	5.4 (10.7)	6.4 (11.9)	2.0 (4.8)	4.0 (8.8)	5.9 (11.0)
	Adjusted Cf. (95% CI)	Ref.		−0.19 (−2.14 to 1.75)	Ref.		1.59 (0.47 to 2.71)	Ref.		4.72 (3.72 to 5.72)	Ref.		2.45 (1.89 to 3.00)	Ref.		2.91 (2.49 to 3.32)
	Cf. (95% CI) after PSM		Ref.	−2.55 (−7.27 to 2.18)		Ref.	1.64 (−2.33 to 5.60)		Ref.	3.50 (−2.19 to 9.19)		Ref.	1.23 (−0.73 to 3.19)		Ref.	2.19 (0.76 to 3.61)
Mechanical ventilation	Events, *n* (%)	17 990 (3.9)	137 (10.0)	126 (9.2)	3936 (1.0)	37 (5.2)	47 (6.7)	13 455 (0.7)	46 (4.1)	77 (6.8)	47 981 (2.1)	140 (9.5)	214 (14.5)	153 876 (3.1)	182 (7.0)	354 (13.6)
	Adjusted RR (95% CI)	Ref.		1.01 (0.84 to 1.21)	Ref.		1.13 (0.82 to 1.55)	Ref.		1.83 (1.43 to 2.34)	Ref.		1.90 (1.64 to 2.19)	Ref.		2.05 (1.84 to 2.27)
	RR (95% CI) after PSM		Ref.	0.92 (0.73 to 1.16)		Ref.	1.27 (0.84 to 1.93)		Ref.	1.67 (1.17 to 2.39)		Ref.	1.53 (1.25 to 1.87)		Ref.	1.95 (1.64 to 2.31)
30‐Day readmission	Events, *n* (%)	27 392 (5.9)	111 (8.1)	229 (16.9)	17 258 (4.6)	52 (7.4)	103 (14.8)	91 076 (4.5)	90 (8.0)	119 (10.7)	144 461 (6.2)	137 (9.5)	175 (12.6)	416 762 (8.8)	260 (10.6)	274 (11.4)
	Adjusted RR (95% CI)	Ref.		2.25 (2.01 to 2.52)	Ref.		1.88 (1.57 to 2.25)	Ref.		1.49 (1.24 to 1.78)	Ref.		1.28 (1.11 to 1.48)	Ref.		1.07 (0.95 to 1.19)
	RR (95% CI) after PSM		Ref.	2.08 (1.68 to 2.57)		Ref.	2.00 (1.46 to 2.75)		Ref.	1.33 (1.02 to 1.72)		Ref.	1.32 (1.07 to 1.63)		Ref.	1.08 (0.92 to 1.26)
1‐Year readmission	Events, *n* (%)	79 579 (17.3)	331 (24.3)	711 (52.5)	62 417 (16.5)	171 (24.3)	331 (47.6)	314 116 (15.6)	276 (24.7)	424 (38.0)	545 852 (23.5)	440 (30.6)	519 (37.3)	1 495 093 (31.5)	826 (33.6)	896 (37.3)
	Adjusted RR (95% CI)	Ref.		2.63 (2.49 to 2.78)	Ref.		2.20 (2.00 to 2.42)	Ref.		1.71 (1.57 to 1.88)	Ref.		1.17 (1.08 to 1.27)	Ref.		1.06 (1.00 to 1.12)
	RR (95% CI) after PSM		Ref.	2.16 (1.95 to 2.41)		Ref.	1.96 (1.68 to 2.28)		Ref.	1.54 (1.36 to 1.75)		Ref.	1.22 (1.10 to 1.35)		Ref.	1.11 (1.03 to 1.20)
2‐Year readmission	Events, *n* (%)	94 679 (20.5)	377 (27.6)	783 (57.8)	83 414 (22.0)	218 (31.0)	377 (54.2)	461 957 (23.0)	348 (31.1)	488 (43.7)	697 008 (30.0)	529 (36.8)	595 (42.7)	1 827 425 (38.6)	983 (40.0)	1009 (42.0)
	Adjusted RR (95% CI)	Ref.		2.49 (2.37 to 2.63)	Ref.		2.04 (1.88 to 2.21)	Ref.		1.52 (1.41 to 1.64)	Ref.		1.12 (1.05 to 1.20)	Ref.		1.01 (0.96 to 1.06)
	RR (95% CI) after PSM		Ref.	2.09 (1.90 to 2.31)		Ref.	1.75 (1.53 to 1.99)		Ref.	1.41 (1.26 to 1.57)		Ref.	1.16 (1.06 to 1.27)		Ref.	1.05 (0.98 to 1.12)

Abbreviations: Cf., regression coefficient; CI, confidence interval; ICU, intensive care unit; IMD, inherited metabolic disorder; PS, propensity score; PSM, propensity score matching; Ref, reference; RR, risk ratio.

30‐day, 1‐year, and 2‐year readmission were increased in all age categories among IMD patients, except the oldest age group of patients over 60 years. Highest risk ratios for 30‐day, 1‐year, and 2‐year readmission were found in children aged 0–9 years with 2.25, 2.63, and 2.49, respectively. Again, results remained robust among propensity score matched cohorts (Table 2). Subgroup analyses exploring differences among specific groups of IMDs revealed similar risk distributions as in the overall analysis (Table S3A–E).

### 3.4 Cause of hospitalization

**FIGURE 4:**
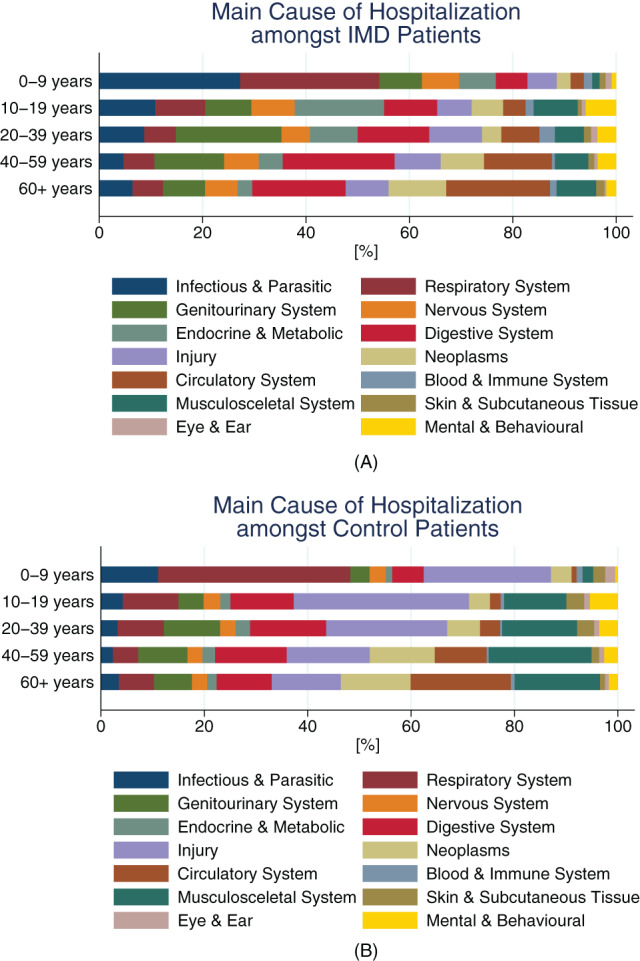
Hospitalization causes for patients with IMD. Shown are causes of hospitalization by ICD‐10‐GM chapter grouping, in percentage of all hospitalizations of selected ICD‐10‐GM chapters per age group for patients with IMD (A) and the overall control population without IMD (B). The main cause of hospitalization was defined as the primary discharge diagnosis, or in the case of IMD being primary—as the secondary discharge diagnosis. IMD, inherited metabolic disorder

When comparing with age‐matched patients without underlying IMD, across all ages those with IMD were more likely to be admitted with a main diagnosis of infectious and parasitic disease, however, main diagnosis of injury and musculoskeletal system, were less common. For children aged 0–19 years, infectious and parasitic causes contributed to the largest proportion of main diagnoses in IMD patients, while respiratory diseases were the most frequent reason of admission among age‐matched non‐IMD controls. In adolescents aged 10–19 and young adults at age 20–39 years, endocrine and metabolic disorders or genitourinary reasons contributed most to hospital admission in patients with IMD, respectively, whereas in the non‐IMD control group, injuries were most prevalent. Above the age of 40, aside from circulatory diseases which was highly prevalent in both IMD patients and controls, digestive system diseases contributed most for IMD patients, and musculoskeletal disorders contributed most for control patients (Figure 4, Table S4A–B).

## 4 DISCUSSION

Our population‐based cohort study has following key findings: First, incidence rates for hospitalization of patients with IMD were highest in young children, level throughout adolescence and adulthood up to the age of 60 years, with an increase thereafter. Second, hospitalized patients with IMD had an increased risk for hospital associated adverse outcomes when compared with other hospitalized patients. While older patients with IMD faced higher risks for in‐hospital adverse outcomes, such as ICU admission and mortality, younger patients with IMD were more prone to being readmitted.

So far, scarce data exist about the lifetime risks for hospitalization of patients with underlying IMD. While clinically stable patients with IMD are usually managed in an interdisciplinary outpatient care setting, metabolic decompensation is a life‐threatening condition requiring emergency admission and in‐hospital work‐up to prevent clinical deterioration. Therefore, incidence rates of metabolic decompensation among patients with IMD may be estimated with high certainty using in‐hospital claims data.

We found highest rates of hospitalization among young children. Children are required to strictly adhere to dietary rules to maintain metabolic stability, with numerous large challenges for patients and caregivers, ranging from issues of palatability of supplements to difficulties in compliance due to patients' neuropsychological profile.^11^, ^12^ Once destabilized by either behavioral aspects or due to acute illness, children must follow precautionary measures, often requiring hospitalization with need of intravenous high‐caloric fluid replacement, the cessation of protein intake and administration of amino acid supplements, and therapy to reduce the amount of toxic metabolites, such as ammonia.^13^ After a steep decrease of incidence rates until the age of 10 years, for AD and CD, there was a marginal increase of hospitalization rates during adolescence with a peak around the age of 15 years. This may be explained by a certain degree of nonadherence to strict diet rules in adolescents growing increasingly independent from their caretakers.^3^, ^11^, ^14^

In Switzerland the newborn screening was introduced in 1965 for phenylketonuria with later expansion of the screening to include further IMDs,^15^ thus patients identified during the early days would not yet be 60 years old during the time period of data collection for this study. Apart from phenylketonuria, which contributed to only a small number of hospitalizations in our analysis, only 227 patients with herein analyzed IMDs had been identified by newborn screening by the end of the study period.^16^ In consequence, most study patients were diagnosed due to clinical manifestation only. The large number of patients in older age groups, supports previous research in this area that onset of IMD in adolescence and adulthood is more frequent than assumed,^3^, ^17^, ^18^ with little or no limitations in life expectancy.

Only few patients with FD were hospitalized, with medium‐chain acyl‐CoA dehydrogenase (MCAD) deficiency being a more recent addition and the only disorder in this group included in the Swiss newborn screening.^19^ Therefore, the number of hospitalizations with FD could be underreported. While incidence rates for FD hospitalization dropped throughout childhood, in contrast to AD and CD, there was a continued decrease even after the age of 20 years with no increase in adulthood. Reasons may include the circumstance that infants need to strictly adhere to a feeding schedule with only short time intervals of fasting, which is no longer required in adults with higher tolerability of longer fasting periods.^20^, ^21^

In line with previous studies,^4^ our results showed strong association of hospitalizations with IMD and a high risk of adverse outcomes and utilization of healthcare. Only limited data is available on mortality in patients with IMD and, particularly, some older studies in neonatal or pediatric ICU settings have suggested high mortality rates of 10.3%–50%.^22^, ^23^, ^24^, ^25^, ^26^ A more recent systematic review assessed mortality rates of about 13% among patients with IMD under the age of 5 years.^4^ As we did a case‐based analysis, findings from our study might not be holistically comparable with previous studies; however, given the far lower mortality rate, our findings indicate a lower risk of death in the early years of life. To better estimate the incremental risk of mortality among patients with IMD as compared with those without IMD, we assessed the risk of mortality in an age‐matched non‐IMD overall inpatient population and—to further increase internal validity—in a pairwise propensity score matched non‐IMD population. Both comparisons revealed an incremental relative risk of about 80%, with, however, an absolute increase of less than 1%. Thus, the risk of mortality is likely to be higher in patients with IMD, however, it may not be as high as assumed in previous studies. Moreover, our findings might be influenced by the study setting, as Switzerland is a high‐income country, and thus findings may not be generalizable to countries with limited health care access. Finally, as the dataset did not include out‐of‐hospital mortality data, including rare cases of sudden infant death, which has been shown to be associated with IMD, we were not able to account for it.^27^

Multiple single‐center studies analyzed the percentage of neonatal or pediatric ICU admissions caused by patients with IMD, with results between 1.1% and 2.6%.^22^, ^23^, ^24^, ^25^, ^26^ In this analysis, children under 10 years with IMD contributed 324 out of 71 287 (0.5%) ICU admissions, again lower than in previous studies. Studies comparing ICU length of stay of IMD patients with non‐IMD controls showed average stays from 3 to 5 days among those with IMD and 6.3 to 7.9 days among controls.^23^, ^25^, ^26^ Our study, however, showed no difference of ICU length of stay when comparing IMD patients to non‐IMD controls. In children, the rate of mechanical ventilation was previously reported at around 65% of intensive care IMD patients,^23^, ^25^ whereas our findings revealed a rate of 38%. Effect size differences are likely to be due to the national full census setting of our study, while most comparable studies were performed in a tertiary center setting and/or a referral center for metabolic diseases or analyzed specifically the neonatal setting. So far, only little is known about the risk for ICU admission and prolonged hospital length of stay among adults with IMD, thus, a comparison is limited.

We further analyzed the cause of hospitalization for patients with IMD. We found an increased proportion of hospitalizations due to infectious and parasitic diseases. This finding seems plausible as infections pose a risk for systemic inflammation‐associated metabolic decompensation with the requirement of a fast and, mostly, intravenously applied therapy to maintain or regain metabolic stability. When looking at common comorbidities of IMDs described in literature,^1^ many patients present with neurological disease possibly because of the accumulation of toxic metabolites. We did not see an increase of neurological diseases as main cause of hospitalization, however, given the chronic course of many neurological diseases (e.g., decrease in cognition), we do not expect neurological diseases to be a major reason for an acute care hospitalization.^28^

Being aware of the current limitations in detecting patients with IMD using the ICD‐10‐GM classification, the introduction of the upgraded version (ICD‐11) and (inter‐) national efforts to create registries for rare diseases^29^, ^30^ will both allow a more precise identification of IMDs in the future. Moreover, it will enrich our findings by providing more specific information on patient numbers and clinical outcomes, as well as medication and laboratory values.

Our data must be interpreted in the context of the study design: IMDs were identified using ICD‐10‐GM codes used for billing purposes, thus, misclassification may be possible. Furthermore, due to the limitations in specificity of ICD‐10‐GM, only a subset of IMDs could be identified, with other large IMDs groups such as disorders of energy metabolism or lysosomal disorders not being included in the analysis. For our national cohort no data exists for the number of patients living with IMDs within Switzerland. Thus, the burden of disease cannot be described per patient, only compared to a general cohort. Given the design of this study, our analysis focused on hospitalized IMD patients only and we were not able to draw any inference on out‐of‐hospital courses. Further, based on the nature of the data, we could not assess whether hospitalization causes were linked to the underlying IMD or not. Finally, clinical parameters and laboratory findings were not available.

However, there are several strengths of note: our analysis was based on nationwide hospital care data with high external validity among high‐income countries, strong power, and a long study period. Additionally, this is the first analysis of epidemiological trends and in‐hospital outcomes among main IMDs groups in a nationwide setting across the lifespan.

## 5 CONCLUSION

This large cohort of nationwide hospitalizations of patients with IMD showed highest hospitalization rates among young children and adults over the age of 60 years. While incremental burden of in‐hospital adverse outcomes was highest among adults, excessive readmission rates were particularly found in children and adolescents. As more (inter‐)national rare disease registries are being established, more granular information on long‐term burden of disease becomes available.

### AUTHOR CONTRIBUTIONS

Stephanie I. Hauser and Alexander Kutz had full access to all the data in the study and take responsibility for the integrity of the data and the accuracy of the data analysis. Concept and design: Hauser, Kutz; acquisition and analysis of data: Hauser, Kutz; statistical analysis: Hauser, Kutz; interpretation of data: Hauser, Kutz; drafting of the manuscript: Hauser, Gregoriano, Kutz; critical revision of the manuscript for important intellectual content: all; obtained funding: Hauser, Kutz, Mueller, Schuetz; supervision: Kutz.

## FUNDING INFORMATION

This study was supported by grants from the Research Council of the Kantonsspital Aarau (Grant Number: 1410.000.172) and the “Hugo und Elsa Isler Fonds” of the Department of Health and Social Affairs of the canton Aargau. The funders had no rule in the design and conduct of this study; collection, management, analysis, and interpretation of the data; preparation, review, or approval of the manuscript; and decision to submit the manuscript for publication.

## CONFLICT OF INTEREST

HK serves as consultant to the Swiss Database for Dosing Medicinal Products in Pediatrics (SwissPedDose), as board member and head of the Collège A (Leading Swiss Children's Hospital) and as associated board member to the Swiss Coordination Group on Rare Disease (kosek). PS received research support paid to the Institution from Thermofischer, bioMerieux, Néstle Health Services and Abbott Nutrition.

## ETHICS STATEMENT

The institutional review board of Northwestern Switzerland (Ethikkommission Nordwest‐ und Zentralschweiz, EKNZ) declared that this study does not fall under the scope of the Human Research Act as data were anonymized before analysis (EKNZ Project‐ID: Req‐2021‐01397). An authorization from the ethical review board was therefore not required.

## Supporting information


**Data S1 Table S1** (A) ICD‐10 codes for inclusion criteria and case rates. (B) ICD‐10 codes of baseline characteristics and comorbidities
**Table S2** Baseline characteristics among hospitalizations with IMD and controls after PSM
**Table S3** (A) Hospital associated outcomes in IMDs from 0 to 9‐year old's, by groups of IMDs with overall, adjusted and propensity score matched control. (B) Hospital associated outcomes in IMDs from 10 to 19‐year old's, by groups of IMDs with overall, adjusted and propensity score matched control. (C) Hospital associated outcomes in IMDs from 20 to 39‐year old's, by groups of IMDs with overall, adjusted and propensity score matched control. (D) Hospital associated outcomes in IMDs from 40 to 59‐year old's, by groups of IMDs with overall, adjusted and propensity score matched control. (E) Hospital associated outcomes in IMDs from 60 to 90‐year old's, by groups of IMDs with overall, adjusted and propensity score matched control
**Table S4** (A) Hospitalization causes for patients with IMD per IMDs group and overall controls. (B) Hospitalization causes for patients with IMD per age group and overall controls

## Data Availability

The data that support the findings of this study are available upon request from the Swiss Federal Statistical Office (Neuchâtel, Switzerland). Restrictions apply to the availability of these data, which were used under license for this study. Data are available as part of the data on “Medizinische Statistik der Krankenhäuser” with the permission of the Swiss Federal Statistical Office, Section Health Services and Population Health.
