# Supplementary material for: Trends and outcomes of children, adolescents, and adults hospitalized with inherited metabolic disorders: A population‐based cohort study
Source: JIMD Rep. 2022 Aug 10;63(6):581–92. doi: 10.1002/jmd2.12320 (PMC9626668; doi:10.1002/jmd2.12320)
Supplement: Supplementary file 1 — Data S1 Table S1 (A) ICD‐10 codes for inclusion criteria and case rates. (B) ICD‐10 codes of baseline characteristics and comorbidities Table S2 Baseline characteristics among hospitalizations with IMD and controls after PSM Table S3 (A) Hospital associated outcomes in IMDs from 0 to 9‐year old's, by groups of IMDs with overall, adjusted and propensity score matched control. (B) Hospital associated outcomes in IMDs from 10 to 19‐year old's, by groups of IMDs with overall, adjusted and propensity score matched control. (C) Hospital associated outcomes in IMDs from 20 to 39‐year old's, by groups of IMDs with overall, adjusted and propensity score matched control. (D) Hospital associated outcomes in IMDs from 40 to 59‐year old's, by groups of IMDs with overall, adjusted and propensity score matched control. (E) Hospital associated outcomes in IMDs from 60 to 90‐year old's, by groups of IMDs with overall, adjusted and propensity score matched control Table S4 (A) Hospitalization causes for patients with IMD per IMDs group and overall controls. (B) Hospitalization causes for patients with IMD per age group and overall controls [file JMD2-63-581-s001.pdf]

Supplementary Information

**Table S1A**	ICD-10 codes for inclusion criteria and case rates	Page 2-3
**Table S1B**	ICD-10 codes of baseline characteristics and comorbidities	Page 4
**Table S2**	Baseline characteristics among hospitalizations with IMD and controls after PSM	Page 5
**Table S3A**	Hospital associated outcomes in IMDs from 0–9-year old's, by groups of IMDs with overall, adjusted and propensity score matched control	Page 6
**Table S3B**	Hospital associated outcomes in IMDs from 10–19-year old's, by groups of IMDs with overall, adjusted and propensity score matched control	Page 7
**Table S3C**	Hospital associated outcomes in IMDs from 20–39-year old's, by groups of IMDs with overall, adjusted and propensity score matched control	Page 8
**Table S3D**	Hospital associated outcomes in IMDs from 40–59-year old's, by groups of IMDs with overall, adjusted and propensity score matched control	Page 9
**Table S3E**	Hospital associated outcomes in IMDs from 60–90-year old's, by groups of IMDs with overall, adjusted and propensity score matched control	Page 10
**Table S4A**	Hospitalization causes for patients with IMD per IMDs group and overall controls	Page 13-14
**Table S4B**	Hospitalization causes for patients with IMD per age group and overall controls	Page 15-16

Table S1A. ICD-10 Codes for inclusion Criteria and case rates

Name	ICD-10 Diagnosis Code	Cases (%)	Code Position
IEM Group: Peptide, Amine and Amino Acid Metabolism Disorders			
Classical phenylketonuria	E700	124 (1.7%)	Any
Other hyperphenylalaninaemias	E701	69 (0.9%)	Any
Disorders of tyrosine metabolism	E702	35 (0.5%)	Any
Albinism	E703	71 (1.0%)	Any
Other disorders of aromatic amino-acid metabolism	E708	14 (0.2%)	Any
Disorder of aromatic amino-acid metabolism, unspecified	E709	2 (<1%)	Any
Maple-syrup-urine disease	E710	43 (0.6%)	Any
Other disorders of branched-chain amino-acid metabolism	E711	185 (2.5%)	Any
Disorder of branched-chain amino-acid metabolism, unspecified	E712	6 (0.1%)	Any
Disorders of amino-acid transport	E720	536 (7.3%)	Any
Disorders of sulfur-bearing amino-acid metabolism	E721	802 (11.0%)	Any
Disorders of urea cycle metabolism	E722	1449 (19.9%)	Any
Disorders of lysine and hydroxylysine metabolism	E723	62 (0.9%)	Any
Disorders of ornithine metabolism	E724	140 (1.9%)	Any
Disorders of glycine metabolism	E725	53 (0.7%)	Any
Other specified disorders of amino-acid metabolism	E728	64 (0.9%)	Any
Disorder of amino-acid metabolism, unspecified	E729	24 (0.3%)	Any
IEM Group : Disorders of fatty acid and ketone body metabolism			
Disorders of fatty-acid metabolism	E713	502 (6.9%)	Any

IEM Group: Disorders of carbohydrate metabolism			
Glycogen storage disease	E740	570 (7.8%)	Any
Disorders of fructose metabolism	E741	686 (9.4%)	Any
Disorders of galactose metabolism	E742	80 (1.1%)	Any
Other disorders of intestinal carbohydrate absorption	E743	829 (11.4%)	Any
Disorders of pyruvate metabolism and gluconeogenesis	E744	90 (1.2%)	Any
Other specified disorders of carbohydrate metabolism	E748	619 (8.5%)	Any
Disorder of carbohydrate metabolism, unspecified	E749	60 (0.8%)	Any

Table S1B. ICD-10 Codes for baseline characteristics (comorbidities) and cause of hospitalization

Name of Group	ICD-10 Diagnosis Code	Code Position: Comorbidities	Code Position: Cause of Hospitalization[1]
Infectious & Parasitic	A00-B99	Any	Primary
Intestinal Infectious Disease	A00-A09	-	Primary
Bacterial Disease	A20-A49	-	Primary
Viral Infections incl. Hepatitis and HIV	B00-B34	-	Primary
Mycoses, Protozoal and others	B35-B89	-	Primary
Other Infectious & Parasitic	A15-A19, A50-A69, B90-B99	-	Primary
Neoplasms	C00-D48	Any	Primary
Malignant Neoplasms	C00-C97	-	Primary
In Situ and Benign Neoplasms	D00-D48	-	Primary
Blood & Immune System	D50-D90	Any	Primary
Anaemias	D50-D64	-	Primary
Other Diseases of Blood	D65-D77	-	Primary
Immune System	D80-D89	-	Primary
Endocrine & Metabolic	E00-E68, E73.1, E73.8, E73.9, E75-E90	Any	Primary
Disorders of Thyroid Gland	E00-E07	-	Primary
Diabetes Mellitus	E10-E14	-	Primary
Hypoglycemia	E15-E16	-	Primary
Other Endocrine Glands	E20-E35	-	Primary
Nutrition Disorders	E40-E68	-	Primary
Metabolic Disorders	E70-E90	-	Primary
Mental & Behavioural	F00-F99	Any	Primary
Nervous System	G00-G99	Any	Primary

[1] For the cause of hospitalization the primary discharge ICD-10 Diagnosis code was used, unless IMD was coded, in which case the secondary coded diagnosis was used.

Name of Group	ICD-10 Diagnosis Code	Code Position: Comorbidities	Code Position: Cause of Hospitalization[1]
Inflammatory Diseases of the Nervous System	G00-G09	-	Primary
Degenerative and Demyelinating Diseases	G10-G37	-	Primary
Episodic and Paroxysmal Disorders	G40-G47	-	Primary
Other Nervous System	G50-G99	-	Primary
Eye & Ear	H00-H95	Any	Primary
Circulatory System	I00-I99	Any	Primary
Heart Disease incl. Hypertensive Disease	I00-I52	-	Primary
Cerebrovascular Disease and Diseases of Arteries	I60-I79	-	Primary
Diseases of Veins and Lymphatic Vessels	I80-89	-	Primary
Respiratory System	J00-J99	Any	Primary
Respiratory Infections	J00-J22	-	Primary
Chronic Lower Respiratory Diseases	J40-J47	-	Primary
Other Respiratory System	J30-J39, J60-J99	-	Primary
Digestive System	K00-K93	Any	Primary
Diseases of Appendix	K35-K38	-	Primary
Hernia	K40-K46	-	Primary
Diseases of Intestines	K50-K64	-	Primary
Diseases of Liver	K70-K77	-	Primary
Disorders of Gallbladder, Biliary Tract and Pancreas	K80-K87	-	Primary
Other Digestive System	K00-K31, K65-K67, K90-K93	-	Primary
Skin & Subcutaneous Tissue	L00-L99	Any	Primary

Name of Group	ICD-10 Diagnosis Code	Code Position: Comorbidities	Code Position: Cause of Hospitalization[1]
Musculosceletal System	M00-M99	Any	Primary
Arthropathies	M00-M25	-	Primary
Systemic Connective Tissue Disorders	M30-M36	-	Primary
Other Muscolosceletal	M40-M99	-	Primary
Genitourinary System	N00-N99	Any	Primary
Urinary Tract Diseases	N00-N39	-	Primary
Disorders of Genital Organs	N40-N99	-	Primary
Injuries	S00-T98	Any	Primary
Other	O00-R99, V01-U99	-	Primary

Table S2 - Baseline characteristics among hospitalizations with IMD and controls after PSM

	IMD Patients	PSM Control	SDiff, %
Number of hospitalizations, n	7'293	7'293	
Number of individual patients, n	4'910	7'228	
**Socio-demographics**			
Age, mean (SD)	42.5 (27.7)	42.5 (27.7)	0.000
Female gender, n (%)	3'684 (50.5)	3'585 (49.2)	0.007
Swiss nationality, n (%)	5'345 (73.3)	5'506 (75.5)	-0.051
**Comorbidities, n (%)**			
Infectious & Parasitic	2'144 (29.4)	2'069 (28.4)	-0.023
Neoplasms	841 (11.5)	796 (10.9)	-0.020
Endocrine & Metabolic	3'833 (52.6)	3'924 (53.8)	0.025
Mental & Behavioural	1'785 (24.5)	1'538 (21.1)	-0.081
Nervous System	1'817 (24.9)	1'802 (24.7)	-0.005
Circulatory System	3'149 (43.2)	3'226 (44.2)	0.021
Respiratory System	1'854 (25.4)	1'758 (24.1)	-0.031
Digestive System	2'588 (35.5)	2'520 (34.6)	-0.020
Musculoskeletal System	1'273 (17.5)	1'371 (18.8)	0.035
Genitourinary System	2'508 (34.4)	2'480 (34.0)	-0.008
Injuries	1'416 (19.4)	1'270 (17.4)	-0.052
Elixhauser comorbidities Index, median (IQR)	2 (0, 3)	1 (0, 3)	-0.007
Hospital Frailty Score			-0.072
< 5 points	5'305 (72.7)	5'503 (75.5)	
5-15 points	1'717 (23.5)	1'593 (21.8)	
>15 points	271 (3.7)	197 (2.7)	

Abbreviations: IMD, Inherited Metabolic Disorder; IQR, Interquartile Range; PSM, Propensity Score Matching; SD, standard deviation; SDiff, standardized difference

Table S3A – Hospital associated outcomes in IMDs from 0–9-year old’s, by groups of IMDs with overall, adjusted and propensity score matched control

		Overall controls (n=462’661)	AD		FD		CD	
			PSM controls (n=660)	IMD cases (n=660)	PSM controls (n=282)	IMD cases (n=282)	PSM controls (n=435)	IMD cases (n=435)
**In-hospital mortality**	events, n (%)	1'225 (0.3)	9 (1.4)	15 (2.3)	0 (0)	2 (0.7)	4 (0.9)	6 (1.4)
	unadjusted RR (95% CI)	Ref.		**8.58 (5.19 to 14.20)**		2.68 (0.67 to 10.67)		**5.21 (2.35 to 11.55)**
	adjusted RR (95% CI)	Ref.		**2.15 (1.26 to 3.66)**		1.60 (0.42 to 6.12)		1.38 (0.63 to 3.02)
	RR (95% CI) after PSM		Ref.	1.67 (0.73 to 3.78)	Ref.	N/A	Ref.	1.50 (0.43 to 5.28)
**Length of hospital stay (d)**	mean (SD)	4.0 (10.1)	8.3 (20.1)	7.7 (14.8)	5.0 (8.1)	5.1 (11.0)	7.4 (15.6)	8.9 (17.1)
	unadjusted Cf. (95% CI)	Ref.		**3.65 (2.88 to 4.43)**		1.07 (-0.11 to 2.26)		**4.91 (3.96 to 5.87)**
	adjusted Cf. (95% CI)	Ref.		1.01 (0.27 to 1.74)		-0.18 (-1.30 to 0.94)		**1.90 (1.00 to 2.80)**
	Cf. (95% CI) after PSM		Ref.	-0.62 (-2.53 to 1.29)	Ref.	0.06 (-1.54 to 1.66)	Ref.	1.59 (-0.60 to 3.78)
**ICU admission**	events, n (%)	70'963 (15.3)	155 (23.5)	149 (22.6)	56 (19.9)	37 (13.1)	113 (26.0)	138 (31.7)
	unadjusted RR (95% CI)	Ref.		**1.47 (1.28 to 1.70)**		0.86 (0.63 to 1.16)		**2.07 (1.80 to 2.37)**
	adjusted RR (95% CI)	Ref.		1.00 (0.85 to 1.17)		**0.71 (0.52 to 0.97)**		**1.38 (1.17 to 1.62)**
	RR (95% CI) after PSM		Ref.	0.96 (0.79 to 1.17)	Ref.	**0.66 (0.45 to 0.97)**	Ref.	1.22 (0.99 to 1.51)
**Length of ICU stay (d)**	mean (SD)	3.2 (11.7)	7.5 (17.8)	5.3 (12.2)	3.7 (8.9)	4.8 (10.9)	6.9 (16.8)	7.1 (20.3)
	unadjusted Cf. (95% CI)	Ref.		1.64 (-1.51 to 4.80)		2.05 (-4.76 to 8.86)		2.47 (-0.50 to 5.44)
	adjusted Cf. (95% CI)	Ref.		-1.18 (-4.15 to 1.79)		-3.39 (-9.79 to 3.01)		1.28 (-1.52 to 4.07)
	Cf. (95% CI) after PSM		Ref.	-2.23 (-7.05 to 2.58)	Ref.	1.65 (-5.31 to 8.62)	Ref.	-1.88 (-8.27 to 4.50)
**Mechanical ventilation**	events, n (%)	17’990 (3.9)	65 (9.9)	45 (6.8)	18 (6.4)	13 (4.6)	49 (11.3)	68 (15.6)
	unadjusted RR (95% CI)	Ref.		**1.75 (1.32 to 2.33)**		1.19 (0.70 to 2.02)		**4.02 (3.23 to 5.00)**
	adjusted RR (95% CI)	Ref.		**0.67 (0.50 to 0.91)**		0.73 (0.43 to 1.25)		**1.67 (1.29 to 2.17)**
	RR (95% CI) after PSM		Ref.	**0.69 (0.48 to 1.00)**	Ref.	0.72 (0.36 to 1.45)	Ref.	1.39 (0.99 to 1.95)
**30-day readmission**	events, n (%)	27’392 (5.9)	69 (10.6)	124 (19.2)	15 (5.3)	31 (11.1)	32 (7.4)	74 (17.3)
	unadjusted RR (95% CI)	Ref.		**3.24 (2.76 to 3.80)**		**1.87 (1.34 to 2.60)**		**2.91 (2.36 to 3.58)**
	adjusted RR (95% CI)	Ref.		**2.37 (2.03 to 2.77)**		**1.80 (1.36 to 2.40)**		**2.34 (1.92 to 2.84)**
	RR (95% CI) after PSM		Ref.	**1.81 (1.38 to 2.38)**	Ref.	**2.08 (1.15 to 3.77)**	Ref.	**2.32 (1.57 to 3.44)**
**1-year readmission**	events, n (%)	79'579 (17.3)	176 (27.0)	350 (54.3)	57 (20.2)	133 (47.5)	104 (24.1)	228 (53.2)
	unadjusted RR (95% CI)	Ref.		**3.15 (2.93 to 3.38)**		**2.75 (2.43 to 3.12)**		**3.08 (2.82 to 3.37)**
	adjusted RR (95% CI)	Ref.		**2.63 (2.42 to 2.85)**		**2.52 (2.22 to 2.85)**		**2.71 (2.46 to 2.99)**
	RR (95% CI) after PSM		Ref.	**2.01 (1.74 to 2.32)**	Ref.	**2.35 (1.81 to 3.06)**	Ref.	**2.20 (1.82 to 2.66)**
**2-year readmission**	events, n (%)	94'679 (20.5)	200 (30.7)	378 (58.6)	66 (23.4)	155 (55.4)	114 (26.5)	250 (58.3)
	unadjusted RR (95% CI)	Ref.		**2.86 (2.68 to 3.05)**		**2.70 (2.43 to 2.80)**		**2.84 (2.62 to 3.08)**
	adjusted RR (95% CI)	Ref.		**2.44 (2.26 to 2.63)**		**2.49 (2.24 to 2.78)**		**2.58 (2.36 to 2.81)**
	RR (95% CI) after PSM		Ref.	**1.91 (1.67 to 2.18)**	Ref.	**2.37 (1.87 to 2.99)**	Ref.	**2.20 (1.85 to 2.63)**

Abbreviations: AD, disorders of peptide, amine and amino acid metabolism; CD, disorders of carbohydrate metabolism; Cf., Regression Coefficient; CI, confidence interval; FD, disorders of fatty acid and ketone body metabolism mellitus; ICU, Intensive Care Unit; IMD, Inherited Metabolic Disorder; PS, Propensity Score; PSM, Propensity Score Matching; PY, person-years; Ref., Reference; RR, Risk Ratio.

Table S3B - Hospital associated outcomes in IMDs from 10–19-year old’s, by groups of IMDs with overall, adjusted and propensity score matched control

		Overall controls (n=379'511)	AD		FD		CD	
			PSM controls (n=343)	IMD cases (n=343)	PSM controls (n=79)	IMD cases (n=79)	PSM controls (n=285)	IMD cases (n=285)
**In-hospital mortality**	events, n (%)	469 (0.1)	4 (1.2)	8 (2.3)	0 (0)	1 (1.3)	2 (0.7)	2 (0.7)
	unadjusted RR (95% CI)	Ref.		**18.87 (9.46 to 37.66)**		**10.24 (1.46 to 71.97)**		**5.68 (1.42 to 22.66)**
	adjusted RR (95% CI)	Ref.		**2.22 (1.01 to 4.89)**		4.23 (0.73 to 24.46)		1.18 (0.24 to 5.78)
	RR (95% CI) after PSM		Ref.	2.00 (0.61 to 6.58)	Ref.	N/A	Ref.	1.00 (0.14 to 7.05)
**Length of hospital stay (d)**	mean (SD)	3.6 (16.9)	4.7 (8.6)	6.5 (12.2)	5.8 (10.6)	9.0 (17.1)	7.1 (10.7)	8.9 (16.3)
	unadjusted Cf. (95% CI)	Ref.		**2.93 (1.14 to 4.71)**		**5.42 (1.70 to 9.14)**		**5.32 (3.36 to 7.28)**
	adjusted Cf. (95% CI)	Ref.		0.40 (-1.37 to 2.18)		2.74 (-0.95 to 6.43)		**1.95 (0.00 to 3.90)**
	Cf. (95% CI) after PSM		Ref.	1.86 (0.27 to 3.45)	Ref.	3.18 (-1.32 to 7.68)	Ref.	1.80 (-0.47 to 4.07)
**ICU admission**	events, n (%)	34'905 (9.2)	60 (17.5)	82 (23.9)	15 (19.0)	12 (15.2)	63 (22.1)	68 (23.9)
	unadjusted RR (95% CI)	Ref.		**2.60 (2.15 to 3.14)**		**1.65 (0.98 to 2.78)**		**2.59 (2.11 to 3.19)**
	adjusted RR (95% CI)	Ref.		**1.61 (1.29 to 2.03)**		0.74 (0.41 to 1.34)		**1.29 (1.00 to 1.65)**
	RR (95% CI) after PSM		Ref.	**1.34 (1.02 to 1.84)**	Ref.	0.80 (0.40 to 1.60)	Ref.	1.08 (0.80 to 1.46)
**Length of ICU stay (d)**	mean (SD)	1.2 (4.5)	2.1 (5.6)	4.5 (13.2)	2.3 (4.6)	7.8 (6.8)	2.9 (5.2)	4.1 (13.3)
	unadjusted Cf. (95% CI)	Ref.		**4.32 (2.55 to 6.09)**		**5.19 (1.50 to 8.87)**		**3.70 (1.76 to 5.64)**
	adjusted Cf. (95% CI)	Ref.		1.26 (-0.33 to 2.85)		2.59 (-0.71 to 5.89)		1.73 (-0.01 to 3.47)
	Cf. (95% CI) after PSM		Ref.	2.74 (-3.42 to 8.91)	Ref.	4.63 (-0.84 to 10.10)	Ref.	1.56 (-3.94 to 7.07)
**Mechanical ventilation**	events, n (%)	3'836 (1.0)	19 (5.5)	23 (6.7)	4 (5.1)	5 (6.3)	19 (6.7)	19 (6.7)
	unadjusted RR (95% CI)	Ref.		**6.47 (4.35 to 9.61)**		**6.10 (2.61 to 14.26)**		**6.43 (4.16 to 9.94)**
	adjusted RR (95% CI)	Ref.		0.89 (0.55 to 1.45)		0.88 (0.38 to 2.05)		**1.54 (0.98 to 2.42)**
	RR (95% CI) after PSM		Ref.	1.21 (0.67 to 2.18)	Ref.	1.25 (0.35 to 4.48)	Ref.	1.00 (0.54 to 1.85)
**30-day readmission**	events, n (%)	17'258 (4.6)	26 (7.7)	68 (20.3)	4 (5.1)	5 (6.4)	24 (8.5)	30 (10.6)
	unadjusted RR (95% CI)	Ref.		**4.46 (3.60 to 5.51)**		1.41 (0.60 to 3.29)		**2.33 (1.66 to 3.27)**
	adjusted RR (95% CI)	Ref.		**2.47 (1.96 to 3.11)**		1.12 (0.55 to 2.28)		**1.41 (1.02 to 1.95)**
	RR (95% CI) after PSM		Ref.	**2.65 (1.73 to 4.05)**	Ref.	1.27 (0.35 to 4.54)	Ref.	1.25 (0.75 to 2.08)
**1-year readmission**	events, n (%)	62'417 (16.5)	67 (19.8)	177 (52.8)	19 (24.1)	34 (43.6)	69 (24.4)	120 (42.4)
	unadjusted RR (95% CI)	Ref.		**3.21 (2.90 to 3.55)**		**2.65 (2.06 to 3.41)**		**2.58 (2.25 to 2.95)**
	adjusted RR (95% CI)	Ref.		**2.49 (2.19 to 2.82)**		**2.04 (1.53 to 2.73)**		**1.92 (1.64 to 2.25)**
	RR (95% CI) after PSM		Ref.	**2.67 (2.11 to 3.39)**	Ref.	**1.81 (1.14 to 2.89)**	Ref.	**1.74 (1.36 to 2.22)**
**2-year readmission**	events, n (%)	83'414 (22.0)	93 (27.4)	193 (57.6)	23 (29.1)	41 (52.6)	88 (31.1)	143 (50.5)
	unadjusted RR (95% CI)	Ref.		**2.62 (2.39 to 2.87)**		**2.39 (1.93 to 2.95)**		**2.30 (2.05 to 2.58)**
	adjusted RR (95% CI)	Ref.		**2.14 (1.91 to 2.40)**		**2.06 (1.62 to 2.61)**		**1.92 (1.68 to 2.19)**
	RR (95% CI) after PSM		Ref.	**2.10 (1.73 to 2.55)**	Ref.	**1.81 (1.21 to 2.70)**	Ref.	**1.63 (1.32 to 2.00)**

Abbreviations: AD, disorders of peptide, amine and amino acid metabolism; CD, disorders of carbohydrate metabolism; Cf., Regression Coefficient; CI, confidence interval; FD, disorders of fatty acid and ketone body metabolism mellitus; ICU, Intensive Care Unit; IMD, Inherited Metabolic Disorder; PS, Propensity Score; PSM, Propensity Score Matching; PY, person-years; Ref., Reference; RR, Risk Ratio.

Table S3C – Hospital associated outcomes in IMDs from 20–39-year old's, by groups of IMDs with overall, adjusted and propensity score matched control

		Overall controls (n=2'015'176)	AD		FD		CD	
			PSM controls (n=574)	IMD cases (n=574)	PSM controls (n=47)	IMD cases (n=47)	PSM controls (n=507)	IMD cases (n=507)
**In-hospital mortality**	events, n (%)	2'649 (0.1)	5 (0.9)	10 (1.7)	0 (0)	2 (4.3)	4 (0.8)	0 (0)
	unadjusted RR (95% CI)	Ref.		**13.25 (7.16 to 24.53)**		**32.38 (8.34 to 125.71)**		N/A
	adjusted RR (95% CI)	Ref.		1.84 (0.97 to 3.48)		**12.38 (3.33 to 46.07)**		N/A
	RR (95% CI) after PSM		Ref.	2.00 (0.69 to 5.81)	Ref.	N/A	Ref.	N/A
**Length of hospital stay (d)**	mean (SD)	3.8 (12.2)	6.5 (10.5)	9.6 (23.9)	4.0 (3.1)	6.4 (8.2)	6.0 (8.6)	7.6 (14.8)
	unadjusted Cf. (95% CI)	Ref.		**5.83 (4.84 to 6.83)**		2.67 (-0.81 to 6.15)		**3.85 (2.79 to 4.91)**
	adjusted Cf. (95% CI)	Ref.		**4.07 (3.08 to 5.06)**		1.62 (-1.83 to 5.08)		2.05 (0.99 to 3.10)
	Cf. (95% CI) after PSM		Ref.	3.08 (0.94 to 5.22)	Ref.	2.40 (-0.14 to 4.94)	Ref.	1.60 (0.11 to 3.09)
**ICU admission**	events, n (%)	138'270 (6.9)	110 (19.2)	153 (26.7)	8 (17.0)	13 (27.7)	84 (16.6)	103 (20.3)
	unadjusted RR (95% CI)	Ref.		**3.88 (3.39 to 4.45)**		**4.03 (2.54 to 6.40)**		**2.96 (2.49 to 3.52)**
	adjusted RR (95% CI)	Ref.		**1.81 (1.52 to 2.16)**		**2.13 (1.23 to 3.69)**		**1.43 (1.17 to 1.75)**
	RR (95% CI) after PSM		Ref.	**1.39 (1.12 to 1.73)**	Ref.	1.63 (0.74 to 3.55)	Ref.	1.23 (0.95 to 1.59)
**Length of ICU stay (d)**	mean (SD)	1.0 (4.3)	3.0 (5.6)	8.8 (27.9)	1.0 (1.2)	1.7 (2.6)	2.1 (4.0)	1.9 (3.8)
	unadjusted Cf. (95% CI)	Ref.		**10.74 (9.40 to 12.08)**		0.68 (-4.33 to 5.69)		1.36 (-0.50 to 3.21)
	adjusted Cf. (95% CI)	Ref.		**7.81 (6.56 to 9.06)**		-2.79 (-7.45 to 1.88)		-0.40 (-2.13 to 1.32)
	Cf. (95% CI) after PSM		Ref.	7.86 (-0.55 to 16.26)	Ref.	1.48 (-1.58 to 4.54)	Ref.	0.24 (-1.65 to 2.12)
**Mechanical ventilation**	events, n (%)	13'455 (0.7)	32 (5.6)	47 (8.2)	2 (4.3)	2 (4.3)	10 (2.0)	28 (5.5)
	unadjusted RR (95% CI)	Ref.		**12.26 (9.32 to 16.14)**		**6.37 (1.64 to 24.74)**		**8.27 (5.77 to 11.86)**
	adjusted RR (95% CI)	Ref.		**1.73 (1.24 to 2.43)**		0.92 (0.22 to 3.90)		**2.07 (1.43 to 2.99)**
	RR (95% CI) after PSM		Ref.	1.47 (0.95 to 2.27)	Ref.	1.00 (0.15 to 6.81)	Ref.	**2.80 (1.37 to 5.70)**
**30-day readmission**	events, n (%)	91'076 (4.5)	48 (8.4)	68 (12.1)	1 (2.1)	5 (11.1)	38 (7.6)	46 (9.1)
	unadjusted RR (95% CI)	Ref.		**2.66 (2.13 to 3.33)**		**2.46 (1.08 to 5.61)**		**2.00 (1.52 to 2.64)**
	adjusted RR (95% CI)	Ref.		**1.61 (1.27 to 2.04)**		2.13 (0.94 to 4.85)		1.30 (0.98 to 1.73)
	RR (95% CI) after PSM		Ref.	**1.43 (1.01 to 2.03)**	Ref.	5.22 (0.63 to 42.98)	Ref.	1.20 (0.80 to 1.81)
**1-year readmission**	events, n (%)	314'116 (15.6)	133 (23.4)	233 (41.3)	10 (21.3)	13 (28.9)	131 (26.0)	178 (35.1)
	unadjusted RR (95% CI)	Ref.		**2.65 (2.40 to 2.92)**		**1.85 (1.17 to 2.93)**		**2.25 (2.00 to 2.53)**
	adjusted RR (95% CI)	Ref.		**1.92 (1.70 to 2.17)**		1.57 (0.97 to 2.56)		**1.52 (1.32 to 1.75)**
	RR (95% CI) after PSM		Ref.	**1.77 (1.48 to 2.11)**	Ref.	1.36 (0.66 to 2.78)	Ref.	**1.35 (1.12 to 1.63)**
**2-year readmission**	events, n (%)	461'957 (23.0)	166 (29.2)	257 (45.6)	14 (29.8)	17 (37.8)	168 (33.4)	214 (42.2)
	unadjusted RR (95% CI)	Ref.		**1.99 (1.81 to 2.17)**		**1.65 (1.13 to 2.39)**		**1.84 (1.66 to 2.04)**
	adjusted RR (95% CI)	Ref.		**1.62 (1.46 to 1.80)**		**1.67 (1.15 to 2.42)**		**1.40 (1.24 to 1.58)**
	RR (95% CI) after PSM		Ref.	**1.56 (1.34 to 1.83)**	Ref.	1.27 (0.71 to 2.26)	Ref.	**1.26 (1.08 to 1.48)**

Abbreviations: AD, disorders of peptide, amine and amino acid metabolism; CD, disorders of carbohydrate metabolism; Cf., Regression Coefficient; CI, confidence interval; FD, disorders of fatty acid and ketone body metabolism mellitus; ICU, Intensive Care Unit; IMD, Inherited Metabolic Disorder; PS, Propensity Score; PSM, Propensity Score Matching; PY, person-years; Ref., Reference; RR, Risk Ratio.

Table S3D – Hospital associated outcomes in IMDs from 40–59-year old's, by groups of IMDs with overall, adjusted and propensity score matched control

		Overall controls (n=2'341'845)	AD		FD		CD	
			**PSM controls (n=785)**	**IMD cases (n=785)**	**PSM controls (n=39)**	**IMD cases (n=39)**	**PSM controls (n=655)**	**IMD cases (n=655)**
**In-hospital mortality**	events, n (%)	20'884 (0.9)	29 (3.7)	80 (10.2)	1 (2.6)	0 (0)	11 (1.7)	6 (0.9)
	unadjusted RR (95% CI)	Ref.		**11.43 (9.28 to 14.07)**		N/A		1.03 (0.46 to 2.28)
	adjusted RR (95% CI)	Ref.		**2.72 (2.15 to 3.45)**		N/A		0.48 (0.22 to 1.05)
	RR (95% CI) after PSM		Ref.	**2.76 (1.83 to 4.17)**	Ref.	N/A	Ref.	0.55 (0.20 to 1.47)
**Length of hospital stay (d)**	mean (SD)	4.7 (8.8)	11.1 (16.4)	14.2 (21.7)	13.1 (22.5)	10.3 (17.0)	7.4 (12.8)	8.1 (11.3)
	unadjusted Cf. (95% CI)	Ref.		**9.50 (8.88 to 10.12)**		**5.63 (2.86 to 8.41)**		**3.40 (2.73 to 4.08)**
	adjusted Cf. (95% CI)	Ref.		**4.36 (3.78 to 4.94)**		0.74 (-1.86 to 3.33)		0.90 (0.26 to 1.53)
	Cf. (95% CI) after PSM		Ref.	**3.08 (1.17 to 4.98)**	Ref.	-2.79 (-11.80 to 6.22)	Ref.	0.69 (-0.62 to 2.00)
**ICU admission**	events, n (%)	259'092 (11.1)	238 (30.3)	311 (39.6)	10 (25.6)	9 (23.1)	118 (18.0)	150 (22.9)
	unadjusted RR (95% CI)	Ref.		**3.58 (3.28 to 3.90)**		**2.09 (1.18 to 3.70)**		**2.07 (1.80 to 2.38)**
	adjusted RR (95% CI)	Ref.		**1.71 (1.52 to 1.92)**		1.08 (0.57 to 2.05)		**1.29 (1.11 to 1.50)**
	RR (95% CI) after PSM		Ref.	**1.31 (1.14 to 1.50)**	Ref.	0.90 (0.41 to 1.97)	Ref.	**1.27 (1.02 to 1.58)**
**Length of ICU stay (d)**	mean (SD)	1.7 (4.8)	6.5 (11.9)	8.1 (12.9)	6.0 (9.3)	2.9 (2.2)	4.7 (13.0)	3.1 (9.1)
	unadjusted Cf. (95% CI)	Ref.		**6.64 (5.92 to 7.37)**		0.04 (-3.89 to 3.97)		**2.05 (0.84 to 3.27)**
	adjusted Cf. (95% CI)	Ref.		**3.08 (2.43 to 3.73)**		-2.69 (-6.19 to 0.81)		**1.18 (0.09 to 2.26)**
	Cf. (95% CI) after PSM		Ref.	1.72 (-0.71 to 4.16)	Ref.	-5.67 (-13.14 to 1.79)	Ref.	-1.40 (-5.21 to 2.40)
**Mechanical ventilation**	events, n (%)	47'981 (2.1)	111 (14.1)	186 (23.7)	3 (7.7)	0 (0)	37 (5.7)	28 (4.3)
	unadjusted RR (95% CI)	Ref.		**11.56 (10.20 to 13.12)**		N/A		**2.09 (1.45 to 3.00)**
	adjusted RR (95% CI)	Ref.		**2.68 (2.27 to 3.15)**		N/A		0.83 (0.58 to 1.19)
	RR (95% CI) after PSM		Ref.	**1.68 (1.35 to 2.07)**	Ref.	N/A	Ref.	0.76 (0.47 to 1.22)
**30-day readmission**	events, n (%)	144'461 (6.2)	77 (10.2)	96 (13.6)	1 (2.6)	3 (7.7)	53 (8.2)	76 (11.7)
	unadjusted RR (95% CI)	Ref.		**2.19 (1.82 to 2.64)**		1.24 (0.42 to 3.67)		**1.88 (1.52 to 2.32)**
	adjusted RR (95% CI)	Ref.		1.20 (0.99 to 1.47)		0.80 (0.27 to 2.39)		**1.42 (1.15 to 1.76)**
	RR (95% CI) after PSM		Ref.	**1.34 (1.01 to 1.77)**	Ref.	2.92 (0.32 to 26.88)	Ref.	**1.42 (1.02 to 1.99)**
**1-year readmission**	events, n (%)	545'852 (23.5)	239 (31.6)	274 (38.9)	11 (29.0)	14 (35.9)	181 (28.1)	231 (35.6)
	unadjusted RR (95% CI)	Ref.		**1.65 (1.51 to 1.81)**		**1.53 (1.00 to 2.32)**		**1.51 (1.36 to 1.68)**
	adjusted RR (95% CI)	Ref.		1.10 (0.98 to 1.23)		1.13 (0.71 to 1.79)		**1.26 (1.12 to 1.40)**
	RR (95% CI) after PSM		Ref.	**1.23 (1.07 to 1.41)**	Ref.	1.24 (0.65 to 2.38)	Ref.	**1.27 (1.08 to 1.49)**
**2-year readmission**	events, n (%)	697'008 (30.0)	280 (37.0)	311 (44.1)	16 (42.1)	17 (43.6)	226 (35.1)	267 (41.1)
	unadjusted RR (95% CI)	Ref.		**1.47 (1.35 to 1.60)**		**1.45 (1.02 to 2.07)**		**1.37 (1.25 to 1.50)**
	adjusted RR (95% CI)	Ref.		**1.06 (0.96 to 1.17)**		1.18 (0.80 to 1.75)		**1.19 (1.08 to 1.31)**
	RR (95% CI) after PSM		Ref.	**1.19 (1.05 to 1.35)**	Ref.	1.04 (0.62 to 1.73)	Ref.	**1.17 (1.02 to 1.35)**

Abbreviations: AD, disorders of peptide, amine and amino acid metabolism; CD, disorders of carbohydrate metabolism; Cf., Regression Coefficient; CI, confidence interval; FD, disorders of fatty acid and ketone body metabolism mellitus; ICU, Intensive Care Unit; IMD, Inherited Metabolic Disorder; PS, Propensity Score; PSM, Propensity Score Matching; PY, person-years; Ref., Reference; RR, Risk Ratio.

Table S3E – Hospital associated outcomes in IMDs from 60–90-year old's, by groups of IMDs with overall, adjusted and propensity score matched control

		Overall controls (n=4'909'425)	AD		FD		CD	
			PSM controls (n=1'276)	IMD cases (n=1'276)	PSM controls (n=55)	IMD cases (n=55)	PSM controls (n=1'271)	IMD cases (n=1'271)
**In-hospital mortality**	events, n (%)	168'793 (3.4)	102 (8.0)	157 (12.3)	4 (7.3)	2 (3.6)	56 (4.4)	38 (3.0)
	unadjusted RR (95% CI)	Ref.		**3.58 (3.09 to 4.14)**		1.06 (0.27 to 4.12)		0.87 (0.64 to 1.19)
	adjusted RR (95% CI)	Ref.		**2.19 (1.88 to 2.55)**		1.01 (0.26 to 3.90)		0.74 (0.54 to 1.00)
	RR (95% CI) after PSM		Ref.	**1.54 (1.21 to 1.95)**	Ref.	0.50 (0.10 to 2.62)	Ref.	0.68 (0.45 to 1.02)
**Length of hospital stay (d)**	mean (SD)	7.5 (10.5)	11.9 (13.5)	16.4 (24.7)	10.1 (10.4)	8.9 (9.1)	8.8 (9.7)	11.1 (13.7)
	unadjusted Cf. (95% CI)	Ref.		**8.91 (8.34 to 9.49)**		1.42 (-1.37 to 4.22)		**3.60 (3.02 to 4.18)**
	adjusted Cf. (95% CI)	Ref.		**4.87 (4.34 to 5.40)**		-0.62 (-3.20 to 1.96)		**1.91 (1.37 to 2.44)**
	Cf. (95% CI) after PSM		Ref.	**4.48 (2.93 to 6.03)**	Ref.	-1.20 (-4.92 to 2.51)	Ref.	**2.33 (1.40 to 3.25)**
**ICU admission**	events, n (%)	673'744 (13.7)	333 (26.1)	499 (39.1)	16 (29.1)	9 (16.4)	219 (17.2)	277 (21.8)
	unadjusted RR (95% CI)	Ref.		**2.85 (2.66 to 3.05)**		1.19 (0.66 to 2.17)		**1.59 (1.43 to 1.76)**
	adjusted RR (95% CI)	Ref.		**1.79 (1.65 to 1.95)**		0.82 (0.44 to 1.51)		**1.22 (1.10 to 1.36)**
	RR (95% CI) after PSM		Ref.	**1.50 (1.34 to 1.68)**	Ref.	0.56 (0.27 to 1.16)	Ref.	**1.26 (1.08 to 1.48)**
**Length of ICU stay (d)**	mean (SD)	2.0 (4.8)	5.5 (11.2)	7.5 (12.7)	2.5 (3.6)	4.5 (5.3)	2.6 (4.2)	3.1 (6.5)
	unadjusted Cf. (95% CI)	Ref.		**6.67 (6.10 to 7.23)**		2.73 (-1.44 to 6.90)		**1.23 (0.46 to 2.00)**
	adjusted Cf. (95% CI)	Ref.		**4.22 (3.70 to 4.73)**		0.71 (-3.10 to 4.52)		0.51 (-0.20 to 1.22)
	Cf. (95% CI) after PSM		Ref.	**2.42 (0.33 to 4.52)**	Ref.	2.45 (-1.97 to 6.86)	Ref.	0.47 (-0.86 to 1.81)
**Mechanical ventilation**	events, n (%)	153'876 (3.1)	125 (9.8)	273 (21.4)	6 (10.9)	2 (3.6)	55 (4.3)	79 (6.2)
	unadjusted RR (95% CI)	Ref.		**6.83 (6.14 to 7.58)**		1.16 (0.30 to 4.52)		**1.98 (1.60 to 2.46)**
	adjusted RR (95% CI)	Ref.		**2.70 (2.39 to 3.06)**		0.46 (0.11 to 1.92)		**1.27 (1.03 to 1.56)**
	RR (95% CI) after PSM		Ref.	**2.18 (1.79 to 2.66)**	Ref.	0.33 (0.07 to 1.58)	Ref.	**1.44 (1.03 to 2.01)**
**30-day readmission**	events, n (%)	416'762 (8.8)	138 (11.8)	144 (12.9)	7 (13.7)	4 (7.6)	120 (9.9)	126 (10.2)
	unadjusted RR (95% CI)	Ref.		**1.46 (1.26 to 1.70)**		0.86 (0.33 to 2.20)		1.16 (0.99 to 1.37)
	adjusted RR (95% CI)	Ref.		**1.09 (0.93 to 1.28)**		0.85 (0.34 to 2.15)		1.05 (0.89 to 1.24)
	RR (95% CI) after PSM		Ref.	1.09 (0.88 to 1.36)	Ref.	0.55 (0.17 to 1.77)	Ref.	1.03 (0.82 to 1.31)
**1-year readmission**	events, n (%)	1'495'093 (31.5)	407 (34.7)	426 (38.1)	20 (39.2)	24 (45.3)	401 (33.0)	446 (36.2)
	unadjusted RR (95% CI)	Ref.		**1.21 (1.12 to 1.30)**		**1.44 (1.07 to 1.93)**		**1.15 (1.07 to 1.24)**
	adjusted RR (95% CI)	Ref.		1.01 (0.93 to 1.09)		**1.43 (1.07 to 1.91)**		**1.09 (1.01 to 1.17)**
	RR (95% CI) after PSM		Ref.	1.10 (0.99 to 1.22)	Ref.	1.15 (0.73 to 1.81)	Ref.	1.10 (0.98 to 1.22)
**2-year readmission**	events, n (%)	1'827'425 (38.6)	471 (40.1)	471 (42.1)	21 (41.2)	26 (49.1)	485 (39.9)	512 (41.5)
	unadjusted RR (95% CI)	Ref.		**1.09 (1.02 to 1.17)**		1.27 (0.97 to 1.67)		**1.08 (1.01 to 1.15)**
	adjusted RR (95% CI)	Ref.		0.95 (0.88 to 1.02)		1.29 (0.99 to 1.68)		1.05 (0.98 to 1.12)
	RR (95% CI) after PSM		Ref.	1.05 (0.95 to 1.16)	Ref.	1.19 (0.78 to 1.83)	Ref.	1.04 (0.95 to 1.14)

Abbreviations: AD, disorders of peptide, amine and amino acid metabolism; CD, disorders of carbohydrate metabolism; Cf., Regression Coefficient; CI, confidence interval; FD, disorders of fatty acid and ketone body metabolism mellitus; ICU, Intensive Care Unit; IMD, Inherited Metabolic Disorder; PS, Propensity Score; PSM, Propensity Score Matching; PY, person-years; Ref., Reference; RR, Risk Ratio.

Table S4A – Hospitalization causes for patients with IMD per IMDs group and overall controls

	AD (n=3'638)	CD (n=3'153)	FD (n=502)	Overall Control (n=10'108'618)
**Infectious & Parasitic, n (%)**	**266 (7.3)**	**195 (6.2)**	**143 (28.5)**	**301'799 (3.0)**
Intestinal Infectious	115 (43.2)	99 (50.8)	116 (81.1)	108'676 (36.0)
Bacterial Disease	106 (39.8)	59 (30.3)	13 (9.1)	127'535 (42.3)
Viral Infections incl. Hepatitis & HIV	23 (8.6)	23 (11.8)	12 (8.4)	31'092 (10.3)
Mycoses, Protozoal and others	15 (5.6)	10 (5.1)	1 (0.7)	18'876 (6.3)
Other Infectious & Parasitic	7 (2.6)	4 (2.1)	1 (0.7)	15'620 (5.2)
**Neoplasms, n (%)**	**242 (6.7)**	**223 (7.1)**	**10 (2.0)**	**992'697 (9.8)**
Malignant Neoplasms	217 (89.7)	178 (79.8)	10 (100.0)	776'385 (78.2)
In situ and Benign Neoplasms	25 (10.3)	45 (20.2)	0 (0.0)	216'312 (21.8)
**Blood & Immune System, n (%)**	**39 (1.1)**	**47 (1.5)**	**0 (0.0)**	**53'226 (0.5)**
Anaemias	23 (59)	26 (55)	n.a.	29'821 (56.0)
Other Diseases of Blood	13 (33)	17 (36)	n.a.	20'351 (38.2)
Immune System	3 (8)	4 (9)	n.a.	3'054 (5.7)
**Endocrine & Metabolic, n (%)**	**110 (3.0)**	**231 (7.3)**	**23 (4.6)**	**179'160 (1.8)**
Disorders of Thyroid Gland	4 (3.6)	5 (2.2)	0 (0.0)	24'858 (13.9)
Diabetes Mellitus	20 (18.2)	27 (11.7)	2 (8.7)	55'818 (31.2)
Hypoglycemia	7 (6.4)	25 (10.8)	5 (21.7)	1'223 (0.7)
Other Endocrine Glands	7 (6.4)	8 (3.5)	6 (26.1)	10'725 (6.0)
Nutrition	16 (14.5)	60 (26.0)	3 (13.0)	50'263 (28.1)
Metabolic Disorders	56 (50.9)	106 (45.9)	7 (30.4)	36'273 (20.2)
**Mental & Behavioural, n (%)**	**82 (2.3)**	**73 (2.3)**	**8 (1.6)**	**196'611 (1.9)**
**Nervous System, n (%)**	**279 (7.7)**	**98 (3.1)**	**24 (4.8)**	**252'854 (2.5)**
Inflammatory Diseases	9 (3.2)	2 (2.0)	0 (0.0)	7'322 (2.9)
Degenerative and Demyelinating	22 (7.9)	14 (14.3)	1 (4.2)	45'017 (17.8)
Episodic and Paroxysmal Disorders	169 (60.6)	51 (52.0)	12 (50.0)	131'104 (51.8)
Other Nervous System	79 (28.3)	31 (31.6)	11 (45.8)	69'411 (27.5)
**Eye & Ear, n (%)**	**15 (0.4)**	**25 (0.8)**	**3 (0.6)**	**75'032 (0.7)**
**Circulatory System, n (%)**	**385 (10.6)**	**382 (12.1)**	**12 (2.4)**	**1'166'094 (11.5)**
Heart Disease incl. Hypertensive	176 (45.7)	282 (73.8)	10 (83.3)	775'768 (66.5)
Cerebrovascular and Arteries	191 (49.6)	82 (21.5)	0 (0.0)	286'087 (24.5)
Veins and Lymphatic Vessels	18 (4.7)	18 (4.7)	2 (16.7)	104'239 (8.9)
**Respiratory System, n (%)**	**209 (5.7)**	**258 (8.2)**	**111 (22.1)**	**682'927 (6.8)**
Respiratory Infections	147 (70.3)	140 (54.3)	91 (82.0)	324'808 (47.6)

	AD (n=3'638)	CD (n=3'153)	FD (n=502)	Overall Control (n=10'108'618)
Chronic Lower Respiratory Diseases	18 (8.6)	52 (20.2)	0 (0.0)	101’722 (14.9)
Other Respiratory System	44 (21.1)	66 (25.6)	20 (18.0)	256’397 (37.5)
**Digestive System, n (%)**	**557 (15.3)**	**379 (12.0)**	**27 (5.4)**	**1’016’861 (10.1)**
Appendix	7 (1.3)	16 (4.2)	6 (22.2)	106’292 (10.5)
Hernia	19 (3.4)	35 (9.2)	0 (0.0)	231’318 (22.7)
Intestines	69 (12.4)	154 (40.6)	5 (18.5)	302’644 (29.8)
Liver	339 (60.9)	21 (5.5)	1 (3.7)	35’019 (3.4)
Gallbladder, Biliary Tract and Pancreas	42 (7.5)	70 (18.5)	4 (14.8)	202’982 (20.0)
Other Digestive System	81 (14.5)	83 (21.9)	11 (40.7)	138’606 (13.6)
**Skin & Subcutaneous Tissue, n (%)**	**19 (0.5)**	**60 (1.9)**	**2 (0.4)**	**134’067 (1.3)**
**Musculoskeletal System, n (%)**	**141 (3.9)**	**214 (6.8)**	**25 (5.0)**	**1’400’826 (13.9)**
Arthropathies	83 (58.9)	98 (45.8)	7 (28.0)	753’325 (53.8)
Systemic Connective Tissue Disorders	4 (2.8)	10 (4.7)	0 (0.0)	15’196 (1.1)
Other Muscoloskeletal	54 (38.3)	106 (49.5)	18 (72.0)	632’305 (45.1)
**Genitourinary System, n (%)**	**366 (10.1)**	**285 (9.0)**	**22 (4.4)**	**694’231 (6.9)**
Urinary Tract Diseases	344 (94.0)	236 (82.8)	20 (90.9)	372’293 (53.6)
Disorders of Genital Organs	22 (6.0)	49 (17.2)	2 (9.1)	321’938 (46.4)
**Injuries, n (%)**	**235 (6.5)**	**235 (7.5)**	**26 (5.2)**	**1'422’224 (14.1)**
**Other, n (%)**	**560 (15.4)**	**410 (13.0)**	**60 (12.0)**	**1’418’525 (14.0)**

Abbreviations: AD, disorders of peptide, amine and amino acid metabolism; CD, disorders of carbohydrate metabolism; FD, disorders of fatty acid and ketone body metabolism mellitus; IMD, Inherited Metabolic Disorder

Table S4B – Hospitalization causes for patients with IMD per age group and overall controls

	Age 0-9 years		Age 10-19 years		Age 20-39 years		Age 40-59 years		Age 60-90 years	
	IMD (n=1'377)	Overall controls (n=462'661)	IMD (n=707)	Overall controls (n=379'511)	IMD (n=1'128)	Overall controls (n=2'015'176)	IMD (n=1'479)	Overall controls (n=2'341'845)	IMD (n=2'602)	Overall controls (n=4'909'425)
**Infectious & Parasitic, n (%)**	**254 (18.4)**	**38'697 (8.4)**	**57 (8.1)**	**14'382 (3.8)**	**70 (6.2)**	**35'267 (1.8)**	**65 (4.4)**	**51'206 (2.2)**	**158 (6.1)**	**162'247 (3.3)**
Intestinal Infectious	214 (84.3)	25'464 (65.8)	43 (75.4)	6'903 (48.0)	33 (47.1)	15'694 (44.5)	7 (10.8)	16'182 (31.6)	33 (20.9)	44'433 (27.4)
Bacterial Disease	7 (2.8)	2'467 (6.4)	5 (8.8)	1'591 (11.1)	18 (25.7)	6'974 (19.8)	44 (67.7)	22'979 (44.9)	104 (65.8)	93'524 (57.6)
Viral Infections incl. Hepatitis & HIV	27 (10.6)	7'413 (19.2)	7 (12.3)	3'595 (25.0)	11 (15.7)	5'289 (15.0)	5 (7.7)	4'915 (9.6)	8 (5.1)	9'880 (6.1)
Mycoses, Protozoal and others	4 (1.6)	1'109 (2.9)	2 (3.5)	658 (4.6)	5 (7.1)	2'042 (5.8)	6 (9.2)	3'754 (7.3)	9 (5.7)	11'313 (7.0)
Other Infectious & Parasitic	2 (0.8)	2'244 (5.8)	0 (0.0)	1'635 (11.4)	3 (4.3)	5'268 (14.9)	3 (4.6)	3'376 (6.6)	4 (2.5)	3'097 (1.9)
**Neoplasms, n (%)**	**25 (1.8)**	**13'842 (3.0)**	**32 (4.5)**	**13'654 (3.6)**	**30 (2.7)**	**68'721 (3.4)**	**115 (7.8)**	**274'283 (11.7)**	**273 (10.5)**	**622'197 (12.7)**
Malignant Neoplasms	23 (92.0)	11'128 (80.4)	28 (87.5)	9'075 (66.5)	17 (56.7)	33'847 (49.3)	91 (79.1)	181'518 (66.2)	246 (90.1)	540'817 (86.9)
In situ and Benign Neoplasms	2 (8.0)	2'714 (19.6)	4 (12.5)	4'579 (33.5)	13 (43.3)	34'874 (50.7)	24 (20.9)	92'765 (33.8)	27 (9.9)	81'380 (13.1)
**Blood & Immune System, n (%)**	**15 (1.1)**	**4'113 (0.9)**	**8 (1.1)**	**2'342 (0.6)**	**24 (2.1)**	**4'402 (0.2)**	**8 (0.5)**	**8'155 (0.3)**	**31 (1.2)**	**34'214 (0.7)**
Anaemias	7 (46.7)	1'645 (40.0)	7 (87.5)	1'330 (56.8)	11 (45.8)	2'029 (46.1)	2 (25.0)	3'450 (42.3)	22 (71.0)	21'367 (62.5)
Other Diseases of Blood	6 (40.0)	2'323 (56.5)	0 (0.0)	951 (40.6)	10 (41.7)	1'796 (40.8)	6 (75.0)	3'512 (43.1)	8 (25.8)	11'769 (34.4)
Immune System	2 (13.3)	145 (3.5)	1 (12.5)	61 (2.6)	3 (12.5)	577 (13.1)	0 (0.0)	1'193 (14.6)	1 (3.2)	1'078 (3.2)
**Endocrine & Metabolic, n (%)**	**66 (4.8)**	**4'273 (0.9)**	**91 (12.9)**	**6'612 (1.7)**	**74 (6.6)**	**30'441 (1.5)**	**64 (4.3)**	**53'776 (2.3)**	**69 (2.7)**	**84'058 (1.7)**
Disorders of Thyroid Gland	0 (0.0)	44 (1.0)	0 (0.0)	254 (3.8)	2 (2.7)	4'435 (14.6)	4 (6.2)	10'718 (19.9)	3 (4.3)	9'407 (11.2)
Diabetes Mellitus	1 (1.5)	1'851 (43.3)	8 (8.8)	3'503 (53.0)	9 (12.2)	4'086 (13.4)	9 (14.1)	12'531 (23.3)	22 (31.9)	33'847 (40.3)
Hypoglycemia	23 (34.8)	304 (7.1)	7 (7.7)	50 (0.8)	5 (6.8)	183 (0.6)	2 (3.1)	271 (0.5)	0 (0.0)	415 (0.5)
Other Endocrine Glands	4 (6.1)	251 (5.9)	3 (3.3)	211 (3.2)	5 (6.8)	1081 (3.6)	4 (6.2)	2'156 (4.0)	5 (7.2)	7'026 (8.4)
Nutrition	7 (10.6)	274 (6.4)	5 (5.5)	840 (12.7)	18 (24.3)	16'888 (55.5)	33 (51.6)	22'895 (42.6)	16 (23.2)	9'366 (11.1)
Metabolic Disorders	31 (47.0)	1'549 (36.3)	68 (74.7)	1'754 (26.5)	35 (47.3)	3'768 (12.4)	12 (18.8)	5'205 (9.7)	23 (33.3)	23'997 (28.5)
**Mental & Behavioural, n (%)**	**8 (0.6)**	**1'775 (0.4)**	**31 (4.4)**	**18'145 (4.8)**	**29 (2.6)**	**39'094 (1.9)**	**49 (3.3)**	**57'761 (2.5)**	**46 (1.8)**	**79'836 (1.6)**
**Nervous System, n (%)**	**67 (4.9)**	**10'920 (2.4)**	**44 (6.2)**	**10'851 (2.9)**	**44 (3.9)**	**32'537 (1.6)**	**92 (6.2)**	**63'158 (2.7)**	**154 (5.9)**	**135'388 (2.8)**
Inflammatory Diseases	0 (0.0)	701 (6.4)	1 (2.3)	446 (4.1)	3 (6.8)	1'217 (3.7)	2 (2.2)	1'954 (3.1)	5 (3.2)	3'004 (2.2)
Degenerative and Demyelinating	6 (9.0)	511 (4.7)	3 (6.8)	652 (6.0)	3 (6.8)	3'605 (11.1)	9 (9.8)	7'661 (12.1)	16 (10.4)	32'588 (24.1)
Episodic and Paroxysmal Disorders	42 (62.7)	8'421 (77.1)	32 (72.7)	7'622 (70.2)	24 (54.5)	18'214 (56.0)	41 (44.6)	32'054 (50.8)	93 (60.4)	64'793 (47.9)
Other Nervous System	19 (28.4)	1'287 (11.8)	8 (18.2)	2'131 (19.6)	14 (31.8)	9'501 (29.2)	40 (43.5)	21'489 (34.0)	40 (26.0)	35'003 (25.9)

	Age 0-9 years		Age 10-19 years		Age 20-39 years		Age 40-59 years		Age 60-90 years	
	IMD (n=1'377)	Overall controls (n=462'661)	IMD (n=707)	Overall controls (n=379'511)	IMD (n=1'128)	Overall controls (n=2'015'176)	IMD (n=1'479)	Overall controls (n=2'341'845)	IMD (n=2'602)	Overall controls (n=4'909'425)
**Eye & Ear, n (%)**	**11 (0.8)**	**6’706 (1.4)**	**4 (0.6)**	**3’728 (1.0)**	**10 (0.9)**	**10’333 (0.5)**	**9 (0.6)**	**20’911 (0.9)**	**9 (0.3)**	**33’354 (0.7)**
**Circulatory System, n (%)**	**24 (1.7)**	**3’381 (0.7)**	**23 (3.3)**	**6’905 (1.8)**	**59 (5.2)**	**42’097 (2.1)**	**181 (12.2)**	**219’949 (9.4)**	**492 (18.9)**	**893’762 (18.2)**
Heart Disease incl. Hypertensive	21 (87.5)	1’685 (49.8)	16 (69.6)	3’728 (54.0)	30 (50.8)	17’811 (42.3)	99 (54.7)	134’681 (61.2)	302 (61.4)	617’863 (69.1)
Cerebrovascular and Arteries	2 (8.3)	737 (21.8)	5 (21.7)	830 (12.0)	22 (37.3)	6’530 (15.5)	71 (39.2)	45’633 (20.7)	173 (35.2)	232’357 (26.0)
Veins and Lymphatic Vessels	1 (4.2)	959 (28.4)	2 (8.7)	2’347 (34.0)	7 (11.9)	17’756 (42.2)	11 (6.1)	39’635 (18.0)	17 (3.5)	43’542 (4.9)
**Respiratory System, n (%)**	**251 (18.2)**	**129’744 (28.0)**	**51 (7.2)**	**36’277 (9.6)**	**49 (4.3)**	**97’011 (4.8)**	**82 (5.5)**	**10’7371 (4.6)**	**145 (5.6)**	**312’524 (6.4)**
Respiratory Infections	208 (82.9)	87’969 (67.8)	28 (54.9)	7’960 (21.9)	27 (55.1)	21’997 (22.7)	40 (48.8)	37’645 (35.1)	75 (51.7)	169’237 (54.2)
Chronic Lower Respiratory Diseases	6 (2.4)	6’297 (4.9)	5 (9.8)	2’466 (6.8)	5 (10.2)	3’576 (3.7)	11 (13.4)	15’100 (14.1)	43 (29.7)	74’283 (23.8)
Other Respiratory System	37 (14.7)	35’478 (27.3)	18 (35.3)	25’851 (71.3)	17 (34.7)	71’438 (73.6)	31 (37.8)	54’626 (50.9)	27 (18.6)	69’004 (22.1)
**Digestive System, n (%)**	**57 (4.1)**	**21’238 (4.6)**	**54 (7.6)**	**41’255 (10.9)**	**111 (9.8)**	**159’599 (7.9)**	**298 (20.1)**	**302’813 (12.9)**	**443 (17.0)**	**491’956 (10.0)**
Appendix	3 (5.3)	5’912 (27.8)	11 (20.4)	23’566 (57.1)	8 (7.2)	37’978 (23.8)	5 (1.7)	24’483 (8.1)	2 (0.5)	14’353 (2.9)
Hernia	1 (1.8)	4’397 (20.7)	1 (1.9)	2’224 (5.4)	7 (6.3)	30’049 (18.8)	19 (6.4)	80’331 (26.5)	26 (5.9)	114’317 (23.2)
Intestines	11 (19.3)	4’748 (22.4)	20 (37.0)	6’067 (14.7)	33 (29.7)	39’830 (25.0)	57 (19.1)	86’086 (28.4)	107 (24.2)	165’913 (33.7)
Liver	19 (33.3)	239 (1.1)	4 (7.4)	378 (0.9)	19 (17.1)	2’227 (1.4)	135 (45.3)	12’815 (4.2)	184 (41.5)	19’360 (3.9)
Gallbladder, Biliary Tract and Pancreas	4 (7.0)	305 (1.4)	3 (5.6)	1’739 (4.2)	20 (18.0)	32’964 (20.7)	44 (14.8)	68’852 (22.7)	45 (10.2)	99’122 (20.1)
Other Digestive System	19 (33.3)	5’637 (26.5)	15 (27.8)	7’281 (17.6)	24 (21.6)	16’551 (10.4)	38 (12.8)	30’246 (10.0)	79 (17.8)	78’891 (16.0)
**Skin & Subcutaneous Tissue, n (%)**	**11 (0.8)**	**8’166 (1.8)**	**4 (0.6)**	**11’670 (3.1)**	**11 (1.0)**	**35’969 (1.8)**	**16 (1.1)**	**32’634 (1.4)**	**39 (1.5)**	**45’628 (0.9)**
**Musculoskeletal System, n (%)**	**13 (0.9)**	**7’110 (1.5)**	**45 (6.4)**	**40’504 (10.7)**	**45 (4.0)**	**157’039 (7.8)**	**89 (6.0)**	**435’372 (18.6)**	**188 (7.2)**	**760’801 (15.5)**
Arthropathies	9 (69.2)	2’949 (41.5)	23 (51.1)	24’840 (61.3)	18 (40.0)	83’292 (53.0)	36 (40.4)	216’427 (49.7)	102 (54.3)	425’817 (56.0)
Systemic Connective Tissue Disorders	0 (0.0)	786 (11.1)	0 (0.0)	318 (0.8)	4 (8.9)	1’509 (1.0)	4 (4.5)	3’459 (0.8)	6 (3.2)	9’124 (1.2)
Other Muscoloskeletal	4 (30.8)	3’375 (47.5)	22 (48.9)	15’346 (37.9)	23 (51.1)	72’238 (46.0)	49 (55.1)	215’486 (49.5)	80 (42.6)	325’860 (42.8)
**Genitourinary System, n (%)**	**77 (5.6)**	**13’095 (2.8)**	**47 (6.6)**	**16’411 (4.3)**	**164 (14.5)**	**118’076 (5.9)**	**186 (12.6)**	**208’294 (8.9)**	**199 (7.6)**	**338’355 (6.9)**
Urinary Tract Diseases	73 (94.8)	11’152 (85.2)	43 (91.5)	6’385 (38.9)	147 (89.6)	46’760 (39.6)	167 (89.8)	103’245 (49.6)	170 (85.4)	204’751 (60.5)
Disorders of Genital Organs	4 (5.2)	1’943 (14.8)	4 (8.5)	10’026 (61.1)	17 (10.4)	71’316 (60.4)	19 (10.2)	105’049 (50.4)	29 (14.6)	133’604 (39.5)
**Injuries, n (%)**	**53 (3.8)**	**85’980 (18.6)**	**35 (5.0)**	**114’275 (30.1)**	**82 (7.3)**	**253’930 (12.6)**	**123 (8.3)**	**351’525 (15.0)**	**203 (7.8)**	**616’514 (12.6)**
**Other, n (%)**	**346 (25.1)**	**101’899 (22.0)**	**143 (20.2)**	**40’204 (10.6)**	**295 (26.2)**	**917’380 (45.5)**	**97 (6.6)**	**137’078 (5.9)**	**149 (5.7)**	**221’964 (4.5)**
